# In-Cell Synthesis of Bioorthogonal Alkene Tag S-Allyl-Homocysteine and Its Coupling with Reprogrammed Translation

**DOI:** 10.3390/ijms20092299

**Published:** 2019-05-09

**Authors:** Saba Nojoumi, Ying Ma, Sergej Schwagerus, Christian P. R. Hackenberger, Nediljko Budisa

**Affiliations:** 1 Institut für Chemie, Technische Universität Berlin, Müller-Breslau-Str. 10, D-10623 Berlin, Germany; saba.nojoumi@tu-berlin.de (S.N.); katrinama2010@gmail.com (Y.M.); 2 Institut für Chemie der Humboldt-Universität zu Berlin, Brook-Taylor-Str. 2, D-12489 Berlin, Germany; schwaga@zedat.fu-berlin.de (S.S.); hackenbe@fmp-berlin.de (C.P.R.H.); 3 Leibniz-Forschungsinstitut für Molekulare Pharmakologie (FMP), Campus Berlin-Buch, Robert-Roessle-Str. 10, D-13125 Berlin, Germany; 4 Chair of Chemical Synthetic Biology, Department of Chemistry, University of Manitoba, 144 Dysart Rd, Winnipeg, MB R3T 2N2, Canada

**Keywords:** biorthogonal conjugations, deallylation/deprotection, direct sulfhydration/transsulfuration pathway, homocysteine, methionine metabolism, non-canonical amino acids, *O*-acetyl-homoserine, reprogrammed translation, S-allyl-homocysteine, selective labelling

## Abstract

In this study, we report our initial results on in situ biosynthesis of S-allyl-l-homocysteine (Sahc) by simple metabolic conversion of allyl mercaptan in *Escherichia* *coli*, which served as the host organism endowed with a direct sulfhydration pathway. The intracellular synthesis we describe in this study is coupled with the direct incorporation of Sahc into proteins in response to methionine codons. Together with *O*-acetyl-homoserine, allyl mercaptan was added to the growth medium, followed by uptake and intracellular reaction to give Sahc. Our protocol efficiently combined the in vivo synthesis of Sahc via metabolic engineering with reprogrammed translation, without the need for a major change in the protein biosynthesis machinery. Although the system needs further optimisation to achieve greater intracellular Sahc production for complete protein labelling, we demonstrated its functional versatility for photo-induced thiol-ene coupling and the recently developed phosphonamidate conjugation reaction. Importantly, deprotection of Sahc leads to homocysteine-containing proteins—a potentially useful approach for the selective labelling of thiols with high relevance in various medical settings.

## 1. Introduction

Canonical amino acid Methionine (Met) is believed to be the most recent addition to the genetic code with the main role of endogenous antioxidants [1] in proteins. Met has very few roles in enzymatic catalytic cycles, whereas in protein folding it behaves similar to the other hydrophobic amino acids [2]. Met contains a unique thioether unit, whose sulphur atom (although often involved in S/π interactions with adjacent aromatic amino acids) can easily be replaced by methylene [3], oxygen [2], selenium [4] and even tellurium [5]. Many non-canonical isosteric analogues and surrogates of Met, which are translationally active, are activated by methionyl-tRNA synthetase (MetRS) with a kinetic turnover similar to those of the native substrate [6]. For this reason, the plasticity of substrate binding in wild-type MetRS can be used to co-translate a relatively large number of analogues and surrogates such as metoxinine [2], ethionine [7], homopropargylglycine [8] and azidohomoalanine [9]. Recently, non-canonical amino acid (ncAA) S-allyl-l-homocysteine (Sahc) was identified as a Met analogue that can be incorporated into proteins in response to AUG sense codons [10]. In 2013, F. Truong [11] used a chemical procedure to synthesise Sahc and proved its incorporation into a GFP variant upon feeding Met-auxotrophic *Escherichia coli* cells. In addition to its translational activity, Sahc is also metabolically active, since it serves as a substrate for Met-adenosyltransferases (MATs) which are crucial enzymes in the biosynthesis of the central metabolite S-adenosylmethionine (SAM) [12,13,14,15]. Met is also the standard starting residue in ribosomal translation, although 60% of these residues are removed by N-terminal processing in *E. coli* [16].

**Scheme 1:**
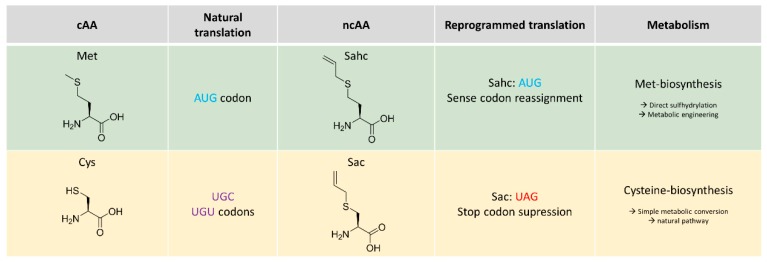
Metabolism and translational activities of S-allyl-l-cysteine (Sac) and S-allyl-l-homocysteine (Sahc). Sahc and Sac differ in their metabolic origin and also have different modes of incorporation into recombinant proteins. While Sahc is the replacement for Met residues in proteins (recognised as a Met analogue), Sac is not aminoacylated by natural Cys translation machinery (i.e., not recognised as Cys analogue). It has recently been incorporated into proteins [22] by using an orthogonal pyrrolysyl tRNA synthetase for in-frame UAG stop codon suppression (cAA = canonical amino acid; ncAA = non-canonical amino acid; blue: AUG sense codon for Met; purple: UGU and UGC sense codons for Cys; red: UAG amber stop codon).

It should be emphasised that Sahc is chemically and structurally similar to S-allylcysteine (Sac) which is naturally abundant in garlic oil [17,18] with a broad range of biological activities. Expectedly, natural chemistry of both Sac and Sahc is similar: Sac is known to be a precursor of allicin (diallylthiosulfinate) which is an allelochemic agent (i.e., defence agent) from garlic (*Allium sativum* L.). Sahc is also abundant in garlic oil [19] and its bioactive properties were documented already in 1955 [20]. Their similarity is plausible, as Sahc differs in chain length by only one carbon atom compared to Sac, while bearing the same functional group. Both can be synthesised in vivo by simple external addition of allyl mercaptan to the growing microbial cultures [21]. However, they are metabolically different as Sac is a derivative of Cys biosynthesis, whereas Sahc is a derivative in Met metabolism (Scheme 1). Finally, they are also different in protein translation. In particular, Sac is only recently incorporated via reprogrammed translation by a dedicated orthogonal pair for in-frame stop codon suppression [22]. On the other hand, Sahc serves as a substrate for endogenous bacterial MetRS [11] and can be used for global substitution of Met residues in proteins.

The choice of model proteins for incorporation studies with our system is of particular importance, as the presence of Met analogues in protein interiors may be detrimental to their functional integrity [23,24,25,26]. For that reason, we have selected a specially designed “hyper stable” GFP construct (GFPhs1-RM) for our initial labelling studies. Compared to the native sequence, it has a much better folding efficiency and particularly better tolerance to ncAA incorporation [27]. Specifically, GFPhs1-RM was optimised to tolerate a completely Met-free protein core, retaining only the N-terminal Met. This protein construct allows the free positioning of Met residues in the entire protein sequence by site-directed mutagenesis and has been successfully used in various settings [28]. In this way, the design of stable target GFP constructs with AUG codons at defined sites become possible.

**Figure A1:**
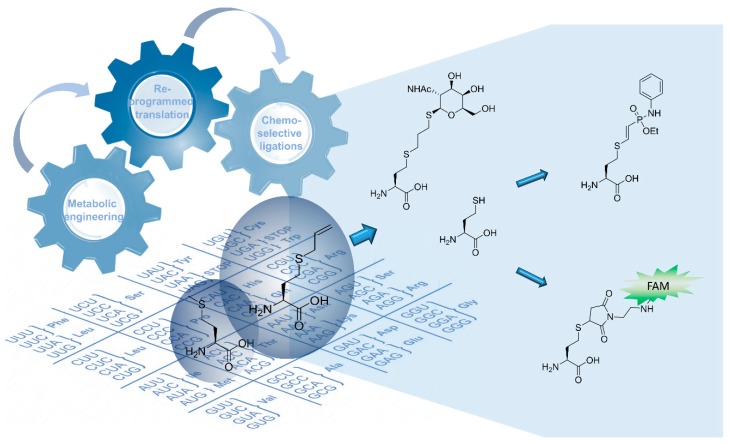
S-allyl-L-homocysteine (Sahc) is a small alkene bioorthogonal tag that can substitute Met in protein translation. We exploited a substrate tolerance of homocysteine synthetases from *Corynebacterium glutamicum* imported into *Escherichia coli* expression host enabling in situ Sahc production. Rewiring Met metabolic pathways towards intracellular noncanonical amino acid synthesis leads to an intracellular accumulation of Sahc and its subsequent incorporation into recombinant proteins, which are accessible to various bioorthogonal transformations.

Significant progress has been made in recent decades in both the residue-specific and site-specific incorporation of non-canonical amino acids (ncAAs) into recombinant proteins [29]. However, the majority of these approaches require the chemical synthesis of the desired ncAA and subsequent delivery to the growth medium, making this process relatively expensive and impractical on a large scale. In this context, we are motivated to couple reprogrammed translation with simple intracellular metabolic conversion of Sahc. To achieve this, we chose to work backwards until the biosynthetic connection to the central metabolism can be made, namely the TCA cycle. We also anticipate that, once fully optimised, such systems will be particularly well suited for the design of a dual modification of thiol groups in proteins provided by various methods, with the potential for spatiotemporal control of their reactivities. Here, we report the first steps of using metabolically engineered *E. coli* [30] capable for in vivo synthesis of Sahc upon feeding the system with the functional allyl mercaptan precursor/handle (Figure A1).

## 2. Results

### 2.1. Metabolic Configuration of E. coli for In Situ Production of Sahc

**Scheme 2:**
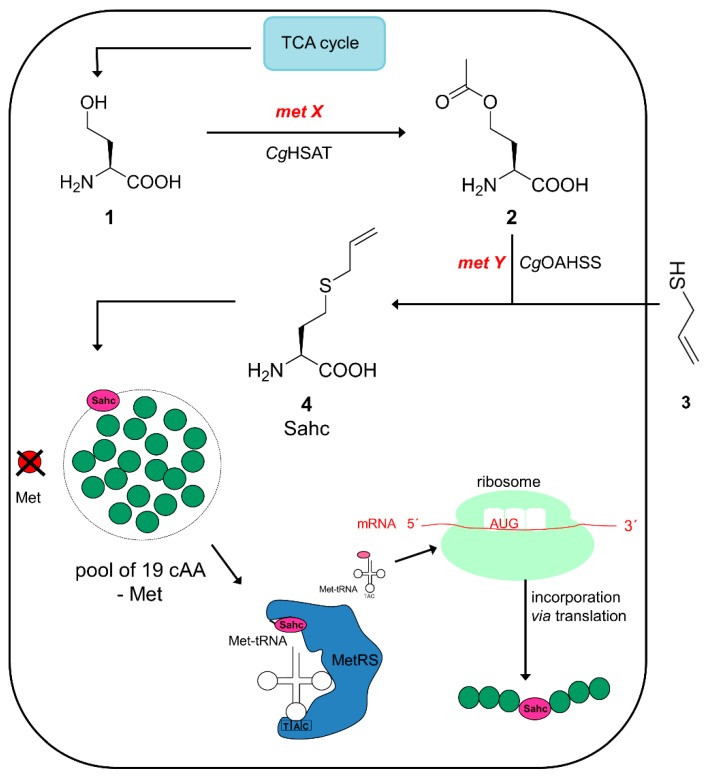
Biosynthesis of S-allyl-L-homocysteine (Sahc) using a re-directed metabolism of homocysteine in *E. coli* B834(DE3). In situ Sahc **4** production is simultaneously coupled with its residue-specific insertion as a response to all AUG codons in the mRNA sequence of recombinant target protein. The *metX* and *metY* genes are imported in *E. coli* [31,34] from methionine metabolism of *C. glutamicum*. *MetX* (*metA* homologue) encodes for homoserine *O*-acetyltransferase activity whereas *metY* (*metB* homologue) encodes *O*-acylhomoserine synthetase (thiolase), which catalyses the second step of the Met biosynthetic pathway of *C. glutamicum* [35].

The metabolic configuration of *E. coli* for in situ production of Sahc is shown in Scheme 2 and is essentially based on the previous works in our group on the metabolic synthesis of azidohomoalanine [31,32]. An additional requirement for our experiments is the auxotrophy of the host cells towards methionine. We used the stable auxotroph *E. coli* B834(DE3) strain (genotype: F^-^ *ompT hsdS_B_(r_B_^−^ m_B_^−^) gal dcm metE*(∆E3)) which bears an inactivated *metE* allele involved in the last step of the Met synthesis pathway under aerobic conditions. Since Sahc is very similar to its canonical counterpart Met, ncAA is recognised and activated by both endogenous MetRS and the ribosomal translation machinery of the host [10,11]. The experimental setup based on these features, which allows the residue-specific (i.e., global) substitution of canonical amino acids with ncAAs, is also known as the selective pressure incorporation (SPI) method [5,33]. By exploiting the substrate tolerance of various cellular systems (uptake, metabolism, and translation apparatus), various ncAAs can be residue-specifically incorporated into target protein sequences. In particular, MetRS could not efficiently differentiate between the canonical substrate Met and Sahc [10,11]. Here, this very feature will be exploited for global substitution of Met residues with Sahc in our model proteins.

To enable our auxotrophic *E. coli* strains to produce Sahc in vivo we engineered the methionine biosynthetic pathway of the host by diverting Met biosynthesis in the metabolism of *E. coli* starting from homoserine **1**, which originates from the TCA cycle towards our desired target analogue Sahc **4**. To achieve this, two genes from *Corynobacterium glutamicum* Met-biosynthetic pathway were cloned and introduced via plasmid vector into our suitably designed *E. coli* B834(DE3) strain deprived of *O*-succinylation [36]. In particular, in the direct sulfhydration pathway of the Met-biosynthesis of *C. glutamicum*, homoserine **1** is converted to *O*-acetyl-l-homoserine (Oahs) **2** by acetyl transfer from acetyl-coenzyme A by the enzyme homoserine acetyltransferase (*Cg*HSAT) which is encoded by gene *metX*. In the next step PLP-dependent enzyme acetyl-l-homoserine sulfhydrylase (*Cg*OAHSS) encoded by *metY* converts Oahs **2** into homocysteine followed by a methylation reaction to give Met. Nucleophilic sulphide produced metabolically serves as a source of sulphur. In addition to the thiol group, a variety of other nucleophiles can be employed as well [32]. On the other hand, Met-biosynthesis is different in *E. coli* [37], where homoserine **1** is first activated by *O*-succinylation in a reaction catalysed by the enzyme MetA, encoded by *metA* gene. Organic sulphide is provided through transsulfuration of *O*-Succinylhomoserine with cysteine (*metB*) to form cystathionine which is further cleaved to homocysteine (*metC*) and finally methylated (*metE*, *metH*) to methionine.

For *E. coli* host engineered this way (see Scheme 2), we expect the in vivo biosynthesis of Sahc to initially start from l-homoserine to *O*-acetyl-l-homoserine catalysed by enzyme *Cg*HSAT. Next, it couples with allyl mercaptan **3** as a nucleophile for intracellular generation of Sahc **4**, catalysed by *Cg*OAHSS. The genes *metY* and *metX* were provided on pBU26′glnS vector (under control of a strong *E. coli* promoter (*glnS*)), while the target gene for model proteins was provided on pQE80L vector (under control of a strong *T5* promoter).

First, *E. coli* B834(DE3) was co-transformed with *metY* bearing vector and a plasmid with the target gene. The equipped engineered host can now produce Sahc **4** out of Oahs **2** and allyl mercaptan **3** as the precursors by direct feeding to the culture medium. Second, to avoid addition of Oahs **2** we used expression strain *E. coli* B834(DE3) equipped with a plasmid containing both *metX* and *metY*. Contrary to our expectations, the performance of the engineered strain compared to parent *E. coli* B834(DE3) (equipped only with *metY*) showed no significant increase in the incorporation levels of Sahc **4** in the target model proteins. Most likely, *metX* does not provide sufficient intracellular *O*-acetyl-l-homoserine levels when compared to the feeding procedure. Obviously, further optimisation and fine tuning of this system is necessary (e.g., by testing different promotor strength combinations and configurations). For this reason, we used *E. coli* B834(DE3) equipped with *metY* gene in all our incorporation experiments.

### 2.2. Expression Experiments and Analytics of Sahc-Incorporation

To investigate the incorporation efficiency of Sahc, GFPhs1-RM was further redesigned with both natural cysteine positions mutated to serine (C70S, C48S). The cysteine-free green fluorescent protein (cfGFPhs1-RM) variants were further changed by site-directed mutagenesis to carry out either one or two Met AUG codon(s) at different positions. In this way, three constructs were generated for conjugation studies. The first construct was C-terminally (His)_6_-tagged cfGFPhs1-RM(M1) bearing M1 on its N-terminus with Q as the penultimate residue. This sequence composition prevents Met1 cleavage by Met-amidotransferase, according to the *N*-end rule [38]. In all incorporation experiments with cfGFPhs1-RM(M1) our *E. coli* B834(DE3) host (equipped with *metY* gene) yielded rather modest levels (about 57%) of N-terminal Met1 substitution with Sahc (see SI for details). The second construct cfGFPhs1-RM(M134) contained only one internal Met-residue introduced by site directed mutagenesis. Its M1 was cleaved by TEV protease along with N-terminal (His)_6_-tag during purification. The expression and incorporation experiments with this variant resulted 86% labelling of the single internal Met-residue (analytical details are provided in SI). In the third construct the cfGFPhs1-RM(M134:M143), two internal Met residues were introduced by site directed mutagenesis at positions 134 and 143. N-terminal M1 in the (His)_6_-tag of cfGFPhs1-RM(M134:M143) was also cleaved by TEV protease during purification. However, the addition of the second labelling site caused a slight drop (~10%) in the labelling power of the system: the expression and incorporation experiments yielded 71% labelling; see Supplementary material and Supplementary Figures S1–S12 for details. The abundance of Sahc in target proteins was calculated from the integral peak intensity in the MS spectra; the combined intensities of the corresponding peaks sum up to 100%, from which each species percentage was calculated and annotated.

The obvious trend is that the incorporation efficiency of Sahc decreased with the number of AUG positions in the sequence. This is further confirmed by our attempts to label proteins encoded by gene sequence with three AUG positions, which has not significantly changed the labelling performance (data not shown, see SI for more information). Taken together, to achieve a high level of analogue incorporation into recombinantly expressed target proteins in our engineered *E. coli* strain, further host optimisation is required (see Section 3).

#### 2.2.1. Thiol-Ene Conjugation Experiments

Sahc is a small-sized bioorthogonal alkene-tag with minimal interference with the protein innate functions, but capable to undergo controlled chemical modifications such as photoinduced thiol-ene coupling. Thiol-ene conjugation was performed only with the constructs with one or two Sahc residues at internal protein positions. Our intention was to test the efficiency of this reaction when only one Sahc was present in the target protein and compare it to the scenario when two Sahc residues were displayed on the same model protein. The synthesised small molecule 2-Acetamido-2-deoxy-β-d-galactopyranosyl-1-thiol (GalNAc-SH) **7** (Figure S13) and thiolated 8PEG hydrogels [39] were chosen for conjugation and immobilisation experiments, respectively. The Sahc-containing proteins cfGFPhs1-RM(134Sahc) **5** and cfGFPhs1-RM(134Sahc:143Sahc) **6** were subjected to a thiol-ene reaction with GalNAc-SH **7**. This specific coupling reaction is initiated by irradiation at 365 nm in aqueous conditions in the presence of photo-initiator for max. 15 min [40].

**Figure 1:**
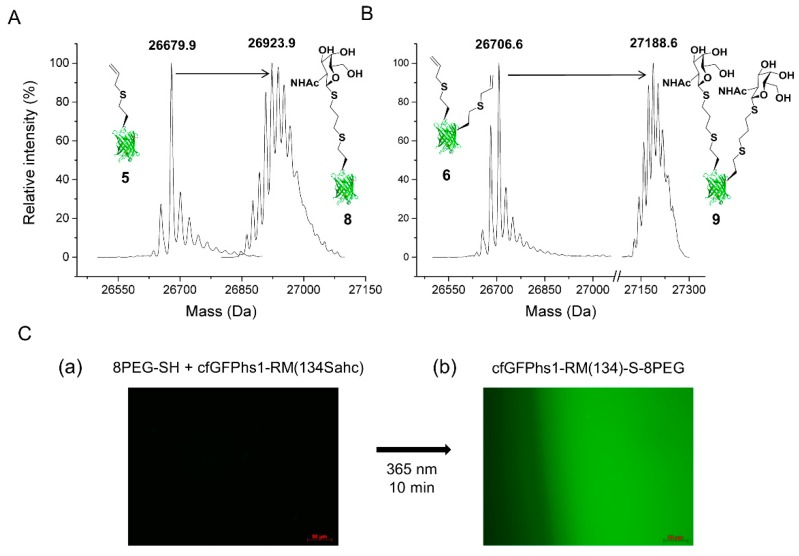
Conjugation reactions with incorporated Sahc as a bioorthogonal handle. (**A**,**B**) Mass spectrometric profiles of (**A**) cfGFPhs1-RM(134Sahc) **5** (calculated MW: 26679.1 Da; detected MW: 26679.9 Da) and (**B**) cfGFPhs1-RM(134Sahc:143Sahc) **6** (calculated MW: 26707.1 Da; detected MW: 26706.6 Da) with the corresponding glycan-conjugates **8** (calculated MW: 26916.1 Da; detected MW: 26923.9 Da) and **9** (calculated MW: 27181.1 Da; measured MW: 27188.6 Da). It should be noted that mass shifts in protein species with conjugated GalNAc **7** (Figure S13) include protonated ([M+7H]^+^ **8** and [M+7H]^+^ **9**) adducts within the error range of our instrument. (**C**) Hydrogel (8PEG-SH hydrogel) with immobilisation of cfGFPhs1-RM(134Sahc): The time interval before irradiation (**a**) and after irradiation (**b**) with 365 nm, was 10 min. The edge of the irradiated hydrogel cfGFPhs1-RM(134)-S-8PEG is visible and shows a contrast between the dark background and the fluorescent gel conjugate.

As expected, the results show that the alkene-bearing constructs cfGFPhs1-RM(134Sahc) **5** and cfGFPhs1-RM(134Sahc:143Sahc) **6** are clearly modified with GalNAc-SH **7**, as confirmed by the observed mass shifts (Figure 1A,B) within the error range of our instrument. In this way, full conversion of Sahc-containing cfGFPhs1-RM constructs into glycan-conjugates was achieved, while the wild-type cfGFPhs1-RM (used as a control sample) remained unchanged. Furthermore, we investigated the immobilising capacities of the Sahc-containing variant cfGFPhs1-RM(134Sahc) variant with the same radical photochemical reaction on 8PEG-SH hydrogels (Figure 1C) resulting in cfGFPhs1-RM(134)-S-8PEG.

The results clearly show the specificity of the reaction by comparing the fluorescence of unreacted hydrogel to the sample exposed to irradiation. As a control, 8PEG-SH hydrogel was subjected to wild-type cfGFPhs1-RM with the same reaction conditions. A successful decoration of the thiolated hydrogels with Sahc-containing proteins can be of particular importance as they are known as biocompatible vehicles for the encapsulation and delivery of proteins for therapeutic purposes [41].

#### 2.2.2. Deprotection of the Sahc Allyl Group

**Figure 2:**
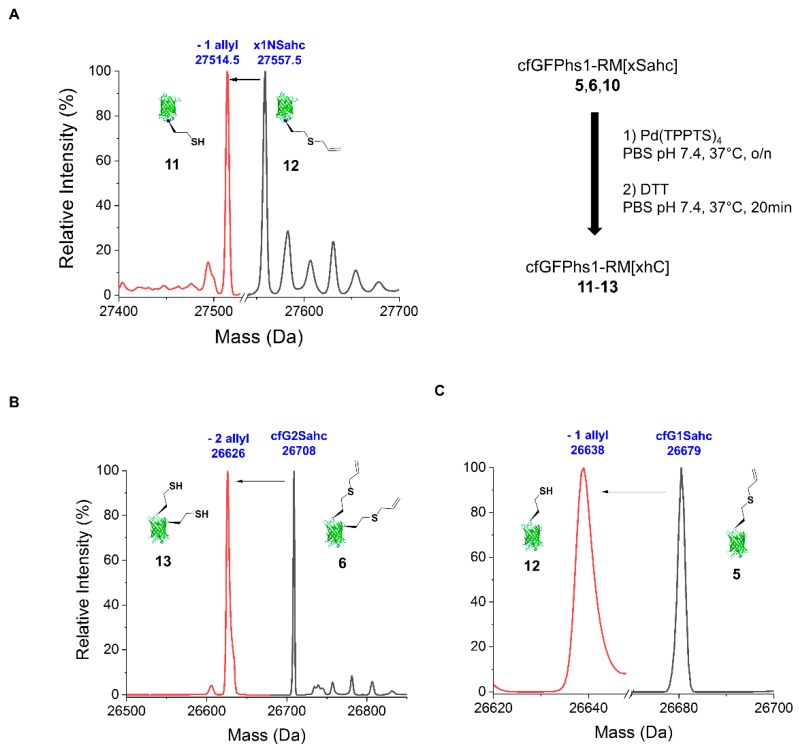
Mass spectrometric profiles of the deallylation reaction catalysed by Pd(TPPTS)_4_ catalysis. (**A**) cfGFPhs1-RM(1Sahc) (x1NSahc) **10** (calculated MW: 27558.99 Da; detected MW: 27557.51 Da) with N-terminal handle, (**B**) cfFPhs1-RM(134Sahc:143Sahc) (cfG2Sahc) **6** (calculated MW: 26707 Da; detected MW: 26707 Da) with two in frame handles, (**C**) cfGFPhs1-RM(134Sahc) (cfG1Sahc) **5** (calculated MW: 26679.14 Da; detected MW: 26679.0 Da) with one in frame handle, were carried out in one-pot reactions with Pd(TPPTS)_4_ catalysts (100 eq.) in PBS pH 7.4 at 37 °C overnight to yield complete deprotected free thiol group (i.e., HCys) bearing constructs **11** (calculated MW: 27517.0 Da; detected MW: 27514.5 Da), **12** (calculated mass: 26638 Da; detected MW: 26638 Da) and **13** (calculated MW: 26627 Da; detected MW: 26626 Da). (o/n—overnight)

Traditionally, allylic handles were used as protecting groups for the SH function in various biological redox environments to protect thiol-containing moieties from side-reactions such as irreversible oxidation via reactive oxygen species [42]. In our previous study, we could demonstrate deallylation reactions [43] by using Pd(0) on *S*-allylcysteine in proteins [22]. To prove the removal capacities for the allylation in Sahc side chains, we used cfGFPhs1-RM(1Sahc) **10**, cfGFPhs1-RM(134Sahc) **5** and cfGFPhs1-RM(134Sahc:143Sahc) **6** as models. The catalyst, a water-soluble palladium complex Pd[PTTPT]_4_, was allowed to react with all Sahc variants, resulting in the deprotection of S-allyl-l-homocysteine according to literature reported protocols [22]. All Sahc-containing proteins were incubated with 100 eq. of Pd(TPPTS)_4_ overnight followed by subsequent quenching with DTT. The use of catalyst in amounts less than 100 eq. (<100 eq.) or shorter reaction times proved to be suboptimal: they led to incomplete removal of the allyl-handle from the Sahc moiety. Optimal reaction conditions as shown in Figure 2 resulted in the 100% efficiency of deprotection (**11**–**13**).

Furthermore, the catalyst itself does not disturb or denature the protein’s structure as shown by fluorescence and MS data. Most probably, the high amounts of catalyst (>100 eq.) are required because of the macromolecular size of the protein carrying the moiety to be deprotected. In this way, the catalyst complexes can collide more efficiently with all solvent-exposed residues of the model protein including the desired Sahc handle. The deprotected constructs (i.e., homocysteine-containing proteins [HCys-Proteins]) were later used for coupling reactions. All relevant chemical syntheses and analytical procedures are provided in supplementary material (see Supplementary Figures S14–S27).

#### 2.2.3. Reaction with FAM-Maleimide and Phosphonamidate Coupling

**Figure 3:**
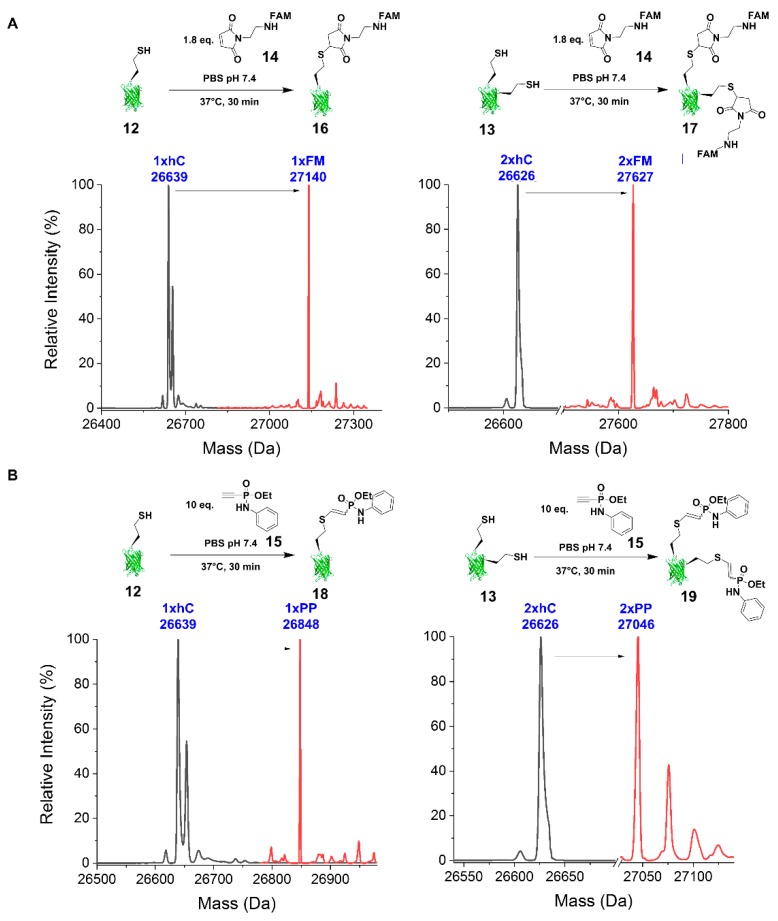
Conjugation reactions of homocysteine-containing deallylated model proteins. (**A**) FAM-maleimide (FM) **14** was conjugated with cfGFPhs1-RM(134HCys) **12** (calculated MW: 26638 Da; detected MW: 26638 Da) and cfGFPhs1-RM(134HCys:143HCys) **13** (calculated MW: 26627 Da; detected MW: 26626 Da) bearing free thiols in PBS pH 7.4 at 37 °C overnight to give cfGFPhs1-RM(134)-S-FM (1xFM) **16** (calculated MW: 27137 Da; detected MW: 27140 Da) [M+3H]^+^ and cfGFPhs1-RM(134:143)-S-FM (2xFM) **17** (calculated MW: 27623 Da; detected MW: 27627 Da) [M+4H]^+^ respectively. (**B**) Phenyl phosphonamidate (PP) **15** was conjugated with cfGFPhs1-RM(134HCys) **12** and cfGFPhs1-RM(134HCys:143HCys) **13** bearing free thiols in PBS pH 7.4 at 37 °C overnight to give cfGFPhs1-RM(134)-S-PP (1xPP) **18** (calculated MW: 26847 Da; detected MW: 26848 Da) [M+H]^+^ and cfGFPhs1-RM(134:143)-S-PP (2xPP) **19** (calculated MW: 27044 Da; detected MW: 27045 Da) [M+H]^+^ respectively.

Deprotection of Sahc yields homocysteine, which shares many chemical properties with cysteine because of the presence of its sulfhydryl group in both molecules. Homocysteine (HCys) differs from cysteine in electrochemical behaviour because of slight differences in hydrophobicity and in structure of their complexes with metals [44]. Since our model proteins were cysteine free, we focused our study on the use of traditional and recently advanced thiol derivatising agents capable for specific reactions with sulfhydryl moieties. For example, phosphonamidate electrophiles are especially suitable for ‘clean’ chemoselective protein conjugation reactions in aqueous systems [45]. Moreover, phosphonamidate conjugates can be further employed for light-induced cleavage strategies [46]. Additionally, this conjugation method requires no catalyst or treatment with additives other than DTT (for ensuring reduced thiols on the protein surface). On the other hand, maleimide-thiol conjugation with sulfhydryl groups is a more conventional conjugation approach with optimal reaction at neutral pH. As shown in Figure 3 the free thiol groups of the deprotected HCys-bearing cfGFPhs1-RM variants (**12**,**13**) reacted almost quantitatively with 6-FAM-maleimide (Lumipore©) (FM) **14** and phenyl phosphonamidate (PP) **15** (synthesised chemically, see [43] and SI for more data). The deprotected constructs bearing free thiols from HCys were incubated either with 1.8 eq. of FAM maleimide (FM) **14** or 10 eq. phenyl phosphonamidate (PP) **15** [45]. Taken together, the efficiency of both reactions proved to be equally good (**16**–**19**) which makes deallylated HCys-containing proteins a useful model for future studies potentially highly relevant for biomedicine. In this context, it may be particularly attractive to use the small physicochemical differences of Sahc versus Sac to find selective reagents with different thiols (e.g., cysteine vs. homocysteine).

## 3. Discussion

### 3.1. Attributes and Perspectives of Sahc as Handle for Selective Protein Conjugation

Alkenes are virtually inert chemical functions in biological systems and thus represent highly interesting bioorthogonal handles to achieve selective protein labelling in cells. When compared with currently available bio-conjugation methods [47,48] (i.e., copper-catalysed azide-alkyne cycloaddition, copper-free reaction, photo-click reaction) [32,49,50,51], the thiol-ene conjugation reaction (also known as “thiol–ene click chemistry”) [52] might be a reasonable alternative. For example, Exner et al. [53] incorporated long chain olefinic amino acids in target GFP and lipase proteins via stop codon suppression and subsequently performed thiol-ene protein conjugation with the anomeric tetrasaccharide. Recently, Met analogue l-homoallylglycine has been incorporated into silk Fibroin, which brought further possible chemistry such as thiol-ene or olefin metathesis to target proteins [54]. In peptide chemistry, it has been already shown that chemically synthesised Sahc is useful in peptide stapling as a functionality utilised by metathesis [10,55], thiol-ene conjugations [52,56,57,58,59] as well as for forming conjugates of hyaluronic acid for cosmetic treatment and preparation methods [60].

In this context, Sahc is a small alkene-tag capable to undergo various chemoselective reactions suitable either for the specific labelling of probes or to study the roles of specific biomolecules in living systems. Here we describe the metabolic synthesis of Sahc and its subsequent incorporation in the recombinant proteins at the Met-positions. We have been able to show that even when the incorporation is not high (i.e., when only a fraction of Sahc is present) proteins are able to meet all the bioorthogonal requirements. Specifically, Sahc is inert to other surrounding molecules in their native environment until specific reaction (thiol-ene coupling and novel phosphonamidate-based conjugation reaction after deallylation) with the desired ligands is initiated.

Of particular interest to Sac and Sahc is that the functional thiol group is masked (protected) by an allyl residue and can be specifically unmasked. Therefore, minimal protection of the thiol functionality should allow; (i) compatibility with biological systems and; (ii) unmasking the thiol functionality at will. Since the specific deprotection (deallylation) of Sahc releases homocysteine thiol functionality, this would allow the tagging and monitoring of critical proteins, particularly those directly involved with homocysteine-related diseases. This should furthermore allow the studies in a natural environment without affecting their structure, function, activity and localisation of target biomolecules. For example, it has been demonstrated that the amide-forming reaction of homocysteine thiolactone with lysine residues induces N-homocysteinylation, which can lead to significant damage to human proteins [61].

In addition, the selective detection of biologically important thiols, such as cysteine and homocysteine, are of great importance for biomedical diagnostics. It is known that such naturally occurring thiols with similar structures can have distinct physiological properties [44]. Clearly, thiols other than cysteine and homocysteine can be selectively distinguished on the basis of the small physicochemical differences, as it is the case with Sahc versus Sac. An important direction of future research would therefore be the development of bioorthogonal tools and approaches that should allow for controlled and targeted detection based on minimal differences between target biomolecules that are so closely related. Here, we face a challenging problem, since the thiol group generally acts as a soft nucleophile in the intracellular redox environment and participates in various reversible and irreversible reactions, making efficient control difficult.

### 3.2. Challenges and Possible Solutions to Intracellular Sahc Production

While we and others [10] have been able to demonstrate Sahc as a useful complement to the tag repertoire for various chemoselective reactions, there is still a challenge with its intracellular production and potency of Met substitutions in the target proteins. The first problem to be considered is the presence of intracellular levels of Met in *E. coli* B834(DE3) *metY* host, which compete efficiently with Sahc for incorporation. In addition, the efficiency of labelling (as judged by the intensities of mass peaks) notably drops upon the increase of AUG codons in the target sequence: cfGFPhs1-RM(134Sahc) (86%), cfGFPhs1-RM(134Sahc:143Sahc) (71%), while further introduction of in-frame AUG codons (cfGFPhs1-RM with 3 Met-residues; see SI for more details) also yielded about 71% of all Met residues replaced with Sahc. Although it is difficult to judge the occupancy (i.e., level of Met to Sahc substitution) for each particular residue, the simple inspection of ESI-MS profiles clearly revealed the trend. On the other hand, the constructs cfGFPhs1-RM(M1) and cfGFPhs1-RM(134Sahc) contain only one Met residue in their sequences. The single Met residue in cfGFPhs1-RM (M1) is located at the N-terminus, whereas cfGFPhs1-RM (134Sahc) contains internal AUG codon (with the N-terminus removed during purification). The initial and internal AUG codons are decoded with different tRNAs and translation in *E. coli* includes N-terminal Met formylation. Therefore, the observed differences in the incorporation efficiencies could be explained by different affinities for Sahc in translation initiation and elongation.

In all of these cases, our metabolically altered host apparently does not provide enough intracellularly synthesised Sahc to compete with the residual Met cellular pool. Therefore, it is important to examine or even identify potential sources of intracellular methionine. A functional salvage pathway as a possible source of residual Met in our auxotrophic B834(DE3) strain should be ruled out as there is no experimental evidence for this phenomenon in *E. coli*, although it is present in some *Bacillus* and *Klebsiella* and *Pseudomonas* bacterial species [62]. In addition, it is reasonable to consider microbial Met recycling by degradation of endogenous proteins or by cleavage of N-terminal Met residues [63] as a significant source of residual Met [64]. Finally, the metabolic configuration of our expression host leaves many important genes involved in Met metabolism and regulation intact. In order to enhance the intracellular production of the desired analogue, the repression of the Met biosynthetic pathway (mediated *inter alia* by gene product of MetJ) [65] can be attenuated. Recently reported strategies to enhance intracellular Met production include *metJ* deletion, overexpression of homoserine [66], Met-regulon de-repression or enhancement of carbon flux for Met-biosynthetic pathways [67] in *E. coli*. Furthermore, residual Met obstacles might be overcome by complete deletion of *metE*, *metH*, *metB* and *metA* genes from the *E. coli* genome. In the long term, designing a microbial strain with allyl mercaptan handle as the only source of sulphur for the cell might be an ideal goal, but would require significant rewiring of the whole bacterial metabolism. All these and other strategies for metabolic engineering [68] should be considered in the design of Met-auxotrophic stains where the production of an analogue should be significantly improved and the intracellular Met concentrations strictly controlled. Finally, only comprehensive metabolomics studies can provide us with important information by monitoring of the intracellular concentrations of all major metabolites and thereby serve as a solid basis for further engineering experiments.

Obviously, metabolic engineering of Sahc in the current state of the art could not compete with the standard method of feeding auxotrophic strains with chemically synthesised compound [11]. It is well known that ncAAs are taken up by the cells via the amino acid transport systems and accumulate up to 70 times within the cell relative to the surrounding medium [69]. This explains the fact of why many ncAAs are translationally active, although they are rather poor substrates for both natural and engineered translation machineries [70]. In particular, the product heterogeneity of our sample is due to the traces of intracellular presence of Met from different sources. However, the incorporation strategy also suffers from competitive rates of Met because the physiochemical and structural difference between Met and Sahc is low. These problems become prominent with the increasing number of Met in our model proteins. In addition to the host metabolic engineering strategies described above, these issues could be further addressed by improving the performance of endogenous MetRS using directed evolution of its substrate specificity. This can also be done with imported heterologous MetRS enzymes from other phylogenetically distant sources as previously described [6]. Next, further optimisation of the fermentation protocol could be accomplished by, for example, by switching to recombinant expression in batch cultures. Finally, by applying the adaptive laboratory evolution strategy [71], the auxotrophic microbial strain can be adapted and grown in the presence of Sahc and similar Met derivatives.

## 4. Materials and Methods

### 4.1. Media and Fermentation Procedure

Previously used new minimal media (NMM) in the context of selective pressure incorporation method SPI [72], has been enhanced (with yeast extract as Met source and higher phosphate buffer concentration for stable pH to avoid anaerobe metabolism) towards better pH balance capacity and is denoted as enhanced new minimal media (ENMM). It contains 7.5 mM (NH_4_)_2_SO_4_, 50 mM K_2_HPO_4_, 22 mM KH_2_PO_4_, 8.5 mM NaCl, 1 mM MgSO_4_, 3.5 g L^−1^ yeast extract (met source), 20 mM d-Glucose, 50 mg L^−1^ 19 canonical amino acids (-Met), 1 mg L^−1^ FeCl_2_, 1 mg L^−1^ CaCl_2_, 10 mg L^−1^ Thiamine, 10 mg L^−1^ Biotin, 10 µg L^−1^ trace elements (CuSO_4_, ZnCl_2_, MnCl_2_, (NH_4_)_2_MoO_4_)). In addition, compared to commercially available Met, yeast extract is more economically efficient as a Met resource for fermentation [73]. For fermentation experiments with *E. coli* B834(DE3) NMM (7.5 mM (NH_4_)_2_SO_4_, 125 mM K_2_HPO_4_, 55 mM KH_2_PO_4_, 8.5 mM NaCl, 1 mM MgSO_4_, 20 mM d-Glucose, 50mg L^−1^ 19 amino acids (-Met), 1 mg L^−1^ FeCl_2_, 1 mg L^−1^ CaCl_2_, 10 mg L^−1^ Thiamine, 10 mg L^−1^ Biotin, 10 µg L^−1^ trace elements (CuSO_4_, ZnCl_2_, MnCl_2_, (NH_4_)_2_MoO_4_) was used as the growth medium [5,74]. 90 mM Met was fed for limiting growth fermentation. The depletion of Met was monitored by measuring a steady cell count at OD_600_ nm.

### 4.2. Strains and Plasmids Used

In vivo biosynthesis of *S*-allyl-l-homocysteine was carried out in strains *E. coli* B834(DE3) (F-*ompT hsdSD*(*rd^-^md-*) *gal dcm metE*) from Novagen Merck Chemicals (Darmstadt, Germany). Vectors pBU26′*glnS-metY* and pBU26′*glnS_metY_metX* were provided by Dr. Y. Ma and Dr. M. L. Di Salvo. Cells transformed with plasmids encoding *metX* and *metY* (pBU26′*glnS_metY_metX*) were grown in ENMM with supplementation of allyl mercaptan and pantothenic acid. Pantothenic acid enhances the biosynthesis of l-homoserine and thus the production of Sahc. In the second approach, the strain *E. coli* B834(DE3) (pBU26′*glnS_metY*, pQE80L_cfGFPhs1-RM) was cultivated in NMM with the addition of allyl mercaptan and *O*-acety-l-homoserine. The target gene construct GFPhs1-RM with either N-terminal or C-terminal (His)_6_-tag and TEV cleavage site was provided by Dr. Nina Bohlke [75]. Plasmid constructs and mutation sites were designed with Geneious (Geneious 7.1.7 (https://www.geneious.com)). cfGFPhs1-RM constructs were expressed on pQE80L vector carrying the T5 promoter/*lac*O element and lambda t^0^ terminator for the target gene. Further features include ColE1 origin, β-lactamase promoter, the β-lactamase resistance cassette, *Lac*I repressor gene and *rrnB* T1 transcription terminator, in the expression host *E. coli* B834(DE3) according to the developed protocol for in vivo synthesis of S-allyl-l-homosysteine described below. Genes *metX* and *metY* were expressed on the vector pBU26′*glnS* carrying the *gln*S’ promoter/terminator system, a kanamycin resistance cassette (pSEVA), p15A *Ori* (pSEVA) and a t^0^ terminator among other features.

### 4.3. Model Protein Cysteine-Free GFPhs1-RM (cfGFPhs1-RM): Generation and Mutagenesis

To omit cross reactivity of endogenous cysteine residues with Sahc **4**, we prepared cysteine-free GFP variant for all incorporation experiments (cfGFPhs1-RM). After cloning the target gene constructs into pQE80L vector, the related genes were designed by site directed mutagenesis which resulted in replacing both cysteines at positions 70 and 84 to serine (C70S, C48S). With cfGFPhs1-RM in hands, we prepared different constructs having various number of AUG codons in the entire gene sequence. The positions D134, E143 in the gene sequence of cfGFPhs1-RM were chosen for site-directed substitutions with either one internal or two internal ATG codons for Sahc **4** in vivo incorporation. Thereby, QuickChange© site directed mutagenesis was used for introducing M134 to give the construct cfGFPhs1-RM(M134) **5** with N-Terminal (His)_6_-tag and TEV-cleavage site. Additionally, for obtaining the double labelled construct, M143 was introduced to give cfGFPhs1-RM(M134:M143) **6** with N-Terminal (His)_6_-tag and TEV-cleavage site, where the initiator M1 is followed by R as penultimate residue retaining N-terminally incorporated Met, which was later cleaved with the (His)_6_-tag. For incorporating Sahc solely at N-terminus the construct cfGFPhs1-RM(M1) **10** was designed with a C-terminal (His)_6_-tag and TEV cleavage site. This single terminally labelled construct carries Q as the penultimate residue at position 2. This amino acid residue ensures that the N-terminally incorporated Met/Sahc residue is retained after translation.

### 4.4. Expression and Purification of cfGFPhs1-RM(1Sahc), cfGFPhs1-RM(134Sahc) and cfGFPhs1-RM(134Sahc:143Sahc)

To start expression experiment 5 mL LB precultures supplemented with Ampicillin (100 µg mL^−1^) and Kanamycin (50 µg mL^−1^) of *E. coli* B834(DE3) (pQE80L_cfGFPhs1-RM(1M), pBU26′*glnS_metY_metX*), (pQE80L_cfGFPhs1-RM(1M), pBU26′*glnS_metY*), *E. coli* B834(DE3) (pQE80L_cfGFPhs1-RM(134M), pBU26′*glnS_metY_metX*), *E. coli* B834(DE3) (pQE80L_cfGFPhs1-RM(134M), pBU26′*glnS_metY*), *E. coli* B834(DE3) (pQE80L_cfGFPhs1-RM(134M:143M), pBU26′*glnS_metY_metX*) and *E. coli* B834(DE3) (pQE80L_cfGFPhs1-RM(134M:143M), pBU26′*glnS_metY*) were prepared and incubated at 37 °C overnight. The following day, 2 mL of overnight culture were added to 1 L ENMM or NMM prepared in a 5 L flask supplemented with Amp (100 µg mL^−1^) and Kan (50 µg mL^−1^) for each strain. The culture was incubated for 8 h at 37 °C shaking at 200 rpm until an OD_600_ of 4.5 was reached. After culture growth was stalled due to Met exhaustion from the medium 0.8 mM allyl mercaptan were added together with 34 µL 1 mM pantothenic acid (total of three addition slots were required in the time intervals of 90 min after the Sahc addition). After 8 h of incubation under this regime, the expression was induced by adding 0.5 mM IPTG and subsequent incubation at 21 °C, 180 rpm, overnight. Cells were harvested the following day at 8000 g, 4 °C, 20 min. Each cell pellet was resuspended in N_A_ buffer (50 mM NaH_2_PO_4_, 300 mM NaCl, 20 mM Imidazole, 5% glycerol, pH 8) and stored at −20 °C. Lysis was performed with a homogeniser followed by centrifugation of the lysate at 18,000 g, 45 min separating cell debris from the lysate. Subsequently, the lysate was filtered with a 45 µm filter tip and purified on a Ni-NTA HiTrapTM FF column at room temperature with a linear gradient. The fluorescent eluate was dialysed in 5 L TEV reaction buffer (50 mM NaH_2_PO_4_, 100 mM NaCl, 0.5 mM Imidazole, 1 mM DTT, pH 8) at 4 °C, overnight. TEV cleavage of the purification trap was performed at room temperature overnight and dialysed in N_A_ buffer at 4 °C overnight. A second purification of the protein mix was performed on a Ni-NTA HiTrapTM FF column at room temperature with a linear gradient to rid of the cleaved purification tag. Purified cfGFPhs1-RM was re-buffered in storage buffer (50 mM NaH_2_PO_4_, 300 mM NaCl, 20% glycerol, pH 8) at 4 °C, overnight. Protein aliquots were stored at −20 °C. Our fermentation and expression experiments reproducibly yield ~10 mg from 1 L of expression culture (NMM, ENMM). All protein variants were purified in the same manner by His-tag Ni-NTA Hi-trap affinity chromatography. Yielded product was stored as 500 µL aliquots in 50 mM NaH_2_PO_4_, 300 mM NaCl, 20% glycerol at −20 °C.

### 4.5. Mass Spectrometry

Sahc-oder Met-containing proteins as well as products of thiol-ene reactions were analyzed by injecting the samples with reverse-phase Agilent 1260 HPLC unit on an Agilent 6530 QTOF instrument (Agilent Technologies, Santa Clara, CA, USA) after external calibration. LC systems and ESI-TOF mass spectrometer were operated as instructed or by qualified persons. Purified Sahc- or Met-containing proteins were subjected to LC systems by injection of 10–15 µL of proteins at a concentration of 0.1 mg/mL. Proteins were separated on a C5 column (Discovery^®^ Bio Wide Pore C5-3, 100 cm × 2.1 mm, 3 µm, Supelco Analytical, Bellefonte, PA, USA) and eluted with a flow rate of 0.3 mL min^−1^. The following gradient was used: A: 0.01% formic acid in H2O; B: 0.01% formic acid in MeCN. 5–95% B 0–22 min. Mass analysis was conducted with Agilent Data acquisition software. Obtained TIC peaks were analyzed with the provided vendor software Mass Hunter^®^ (Agilent Technologies, Santa Clara, CA, USA) for a range of 10 kDa to 40 kDa.

For deallylation and FAM maleimide/phosphonamidate conjugation products a Waters H-class instrument equipped with a quaternary solvent manager, a Waters sample manager-FTN, a Waters PDA detector and a Waters column manager with an Acquity UPLC protein BEH C4 column (300 Å, 1.7 µm, 2.1 mm × 50 mm) were used. Proteins were eluted with a flow rate of 0.3 mL min^−1^. The following gradient was used: A: 0.01% FA in H_2_O; B: 0.01% FA in MeCN. 5–95% B 0–6 min. Mass analysis was conducted with a Waters XEVO G2-XS QTof analyser (Waters, Milford, MA, USA). Proteins were ionised in positive ion mode applying a cone voltage of 40 kV. The resulting spectrum was deconvoluted with the MassLynx^®^ software (Waters, Milford, MA, USA) to obtain the molecular weight.

### 4.6. Deprotection Reactions

3,3′,3″-Phosphanetriyl-Tris(benzenesulfonic acid) trisodium salt (TPPTS) (6 eq., 15.2 mg, 26.8 μmol) was dissolved in 250 μL of PBS pH 7.4. Palladium (II) acetate (1.0 mg, 4.45 μmol) was added to generate the pale brown 17.8 mM Pd(TPPTS)_4_. To cfGFPhs1-RM(134Sahc) **5** (1.78 nmol,) in 36.3 μL PBS pH 7.4 was added the previous solution of Pd(TPPTS)_4_ (17.8 mM). The deprotection was carried out with either 10 eq. (17.8 nmol, 0.1 μL) or 100 eq. (178 nmol, 1 μL) of Pd(TPPTS)_4_. The mixtures were shaken at 37 °C overnight. Next, a large excess of Dithiothreitol (DTT) (1000 eq., 1.78 μmol, 274 μg) was added and the solution was kept at 37 °C for 20 min. Subsequently the mixtures were spin filtered eight times with Amicon^®^ Ultra centrifugal filters (MWCO: 10 kDa) in order to remove the complexed Pd and the DTT. Full deprotection could be achieved with 100 eq. Pd(TPPTS)_4_. The product cfGFPhs1-RM(hC) was analysed by ESI QToF MS. To cfGFPhs1-RM (134Sahc:143Sahc) **6** (1.78 nmol) in 36.3 μL PBS pH 7.4 was added the previous solution of Pd(TPPTS)_4_ (17.8 mM). The deprotection was carried out with either 10 eq. (17.8 nmol, 0.1 μL) or 100 eq. (178 nmol, 1 μL) of Pd(TPPTS)_4_. The mixtures were shaken at 37 °C overnight. Next, a large excess of Dithiothreitol (DTT) (1000 eq., 1.78 μmol, 274 μg) was added and the solution was kept at 37 °C for 20 min. Subsequently the mixtures were spin filtered eight times with Amicon^®^ Ultra centrifugal filters (MWCO: 10 kDa) in order to remove the complexed Pd and the DTT. Full deprotection could be achieved with 100 eq. Pd(TPPTS)_4_. The product cfGFPhs1-RM(2hC) was analysed by ESI QToF mass spectrometry.

### 4.7. Conjugation Reactions

#### 4.7.1. Thiol-Ene Conjugations

To verify that the thiol-ene reaction is suitable for labelling the alkene-bearing cfGFPhs1-RM(134Sahc) **5** and cfGFPhs1-RM(134Sahc:143Sahc) **6** and wild-type cfGFPhs1-RM as control; GalNAc-SH **7** was used as a conjugation partner. A reaction mixture containing 30 µL of 1 M Tris-HCl pH 6.8 (120 mM), 5 µL of 100 mM TCEP (2 mM) was prepared in 120 µL of H_2_O. In a different microtube, a solution of 10 mM photoinitiator I2959 containing 50% DMSO in water was prepared. Then, 62.5 µL of I2959 were added to 250 µL of reaction buffer immediately before conjugation. Next, 2.5 µL of reaction buffer-photoinitiator mix were added to 20 µL of cfGFPhs1-RM(134Sahc) **5** or cfGFP-hs1(134Sahc:143Sahc) **6** (2 mg mL^−1^). The protein mix was incubated for 10 min. GalNAc-SH **7** 250x substrate solution was prepared with 10 µL of 100 mM TCEP (10 mM), 90 µL of 25 mM GalNAc-SH **7**. Subsequently, 16.8 µL of this solution was added to the reaction mixture and incubated at room temperature for another 10 min. The reaction mixture was transferred to an MS glass vial. Samples were placed on a UV LED lamp (365 nm, 1 W) built in our lab and irradiated for 5 min. Next, the reaction sample was transferred to a mass spectrometer vial inlay and diluted with 20 µL 100 mM Tris-HCl. The protein solution was adjusted to 0.2 mg mL^−1^ in 10 mM Tris-HCl buffer. Both samples were then analysed by mass spectrometry. The sample was injected into an ESI-MS with reverse-phase Agilent 1260 HPLC unit on an Agilent 6530 QTOF instrument (Agilent, Santa Clara, Ca, USA) after external calibration. Obtained TIC peaks were deconvoluted for a range of 10 kDa to 40 kDa with the error ranges ± 5–10 Da.

#### 4.7.2. 8PEG-SH Gel Thiol-Ene Reaction—Immobilisation of cfGFPhs1-RM(134Sahc) on Hydrogels

For immobilisation of GFP on 8PEG-SH gels the thiol-ene reaction was performed under the following conditions: First, 8PEG-SH gels of different sizes and thickness were pre-treated with DTT (1 mM) with subsequent multiple washing of the gel with a phosphate buffer to obtain free thiol residues for the following thiol-ene reaction. The protein mix (cfGFPhs1-RM(134Sahc) **5** in reaction solution containing photo-initiator I2959) was prepared and added to the pre-treated 8PEG-SH gel by placing a drop of the protein mix on the gel prepared on a glass slide. After several washing steps and irradiated with 365 nm UV light for 15 min after an incubation for 30 min at RT in order to test penetration of cfGFPhs1-RM(134Sahc) **5** through the gel by diffusion. The protein-gel preparation was covered with a cover slide and incubated for 10 min at room temperature in the dark followed by 10 min UV light irradiation at 365 nm by a standard UV lamp. Subsequently, the gel was washed for 30 min in 100 mM Tris-HCl buffer solution for 30 min. Then, the washed cfGFPhs1-RM(134)-S-8PEG gel was prepared on a fresh glass slide for fluorescence microscopy. The control reaction to exclude hydrogel embedding GFP by trapping, wild type GFPhs1-RM with Met at position 134 was put through the same procedure. Our negative control hydrogel exhibited no fluorescence.

#### 4.7.3. FAM-Maleimide

6-FAM-maleimide **14** (498 Da) was purchased from Lumiprobe© and put to reaction with 45 µM deprotected Sahc bearing protein cfGFPhs1-RM(134hC) **12** or cfGFPhs1-RM(134hC,143hC) **13**. 3 eq. were put to reaction (0.5 mg in 500 µL in DMSO, 1 nmol μl^−1^, 0.5 µL) at 37 °C and incubated in PBS overnight. 6-FAM maleimide (FM) **14** (1.8 eq., 0.5 mg in 500 µL DMSO, 1 nmol µL^−1^, 0.5 µL) was added to cfGFPhs1-RM(hC) **12** (0.28 nmol/10 µL in PBS pH 7.4) and incubated at 37 °C for 30 min. Subsequently the mixture was spin filtered five times with Amicon^®^ Ultra centrifugal filters (MWCO: 10 kDa) in order to remove the excess reagent. The product cfGFPhs1-RM(hC)-FM **16** was analysed by ESI QToF MS. 6-FAM maleimide **14** (1.8 eq., 0.5 mg in 500 µL DMSO, 1 nmol µL^−1^, 0.5 µL) was added to cfGFPhs1-RM(2hC) **13** (0.28 nmol/10 µL in PBS pH 7.4) and incubated at 37 °C for 30 min. Subsequently the mixture was spin filtered five times with Amicon^®^ Ultra centrifugal filters (MWCO: 10 kDa) in order to remove the excess reagent. The product cfGFPhs1-RM(2hC)-2FM **17** was identified and examined by Waters XEVOG2-XS ESI QToF analyser.

#### 4.7.4. Phenyl Phosphonamidate

Phenyl-phosphonamidate **15** (209 Da) was synthesised and put to reaction with 45 µM -deprotected Sahc bearing protein cfGFPhs1-RM(134hC) **12** or cfGFPhs1-RM(134hC,143hC) **13** (6 nmol μL^−1^, 0.5 µL, overnight) at 37 °C in PBS overnight. Q-Tof ESI/MS are data shown in SI. Phenyl phosphonamidate (PP) **15** (10 eq., 6 nmol µL^−1^, 0.5 µL) was added to cfGFPhs1-RM(hC) **12** (0.28 nmol/10 µL in PBS pH 7.4) and incubated at 37 °C overnight. Subsequently the mixture was spin filtered five times with Amicon^®^ Ultra centrifugal filters (MWCO: 10 kDa) in order to remove the excess reagent. The product cfGFPhs1-RM(hC)-PP **18** was analysed by ESI QToF MS. Phenyl phosphonamidate (PP) **15** (10 eq., 6 nmol µL^−1^, 0.5 µL) was added to cfGFPhs1-RM(2hC) **13** (0.28 nmol/10 µL in PBS pH 7.4) and incubated at 37 °C overnight. Subsequently the mixture was spin filtered five times with Amicon^®^ Ultra centrifugal filters (MWCO: 10 kDa) in order to remove the excess reagent. The product cfGFPhs1-RM(2hC)-2PP **19** was identified and examined by Waters XEVOG2-XS ESI QToF mass spectrometer.

## 5. Conclusions

Noncanonical amino acid S-allyl-l-homocysteine (Sahc) is a methionine analogue that can be incorporated into proteins in response to AUG sense codons in related auxotrophic bacterial strains. Sahc is chemically and structurally similar to S-allylcysteine (Sac) which is part of well-known natural product—garlic oil. Both can be synthesised in vivo by simple external addition of allyl mercaptan to the growing microbial cultures. However, they are metabolically different as Sac is a derivative of Cys biosynthesis, whereas Sahc is a derivative of the Met metabolism.

Sahc side chain is a small noninvasive alkene-tag capable of participating in various chemoselective bioorthogonal reactions. For example, Sahc-containing proteins undergo photo-induced thiol-ene coupling (also known as “thiol–ene click chemistry”) with related ligands or even hydrogels resulting in precisely labeled protein products. Furthermore, Sahc allyl moiety can be specifically deprotected via Pd-catalysed deallylation reaction. Resulting protein constructs contain homocysteine, which takes part with its thiol group in various and specific chemoselective reactions such as maleimide and phosphonamidate ligations.

Reprogrammed translation can be coupled with a simple intracellular metabolic conversion of Sahc. However, we conclude that our metabolically altered host is still not powerful enough to produce intracellularly synthesised Sahc to the levels that would enable efficient competition with residual intracellular Met resources. We discussed and proposed strategies for systematic cell engineering that should result in metabolic prototypes that can compete with the standard method of feeding auxotrophic strains with chemically synthesised noncanonical amino acids in reprogramed translation. 

## Supplementary Materials

Supplementary materials can be found at https://www.mdpi.com/1422-0067/20/9/2299/s1.

## Abbreviations

Aha	azidohomoalanine
cAA	canonical amino acid
*Cg*	*Corynobacterium glutamicum*
Cys	cysteine
cfGFP	cysteine-free green fluorescent protein
*E. coli*	*Escherichia coli*
GalNAc	galactosamineacetate
HCys	l-homocysteine
Met	l-methionine
MetRS	methionyl-tRNA synthetase
ncAA	non-canonical amino acid
Oahs	*O*-acetyl-l-homoserine
Sac	S-allyl-l-cysteine
Sahc	S-allyl-l-homocysteine
SPI	Selective Pressure Incorporation

## References

[1] Schindeldecker M., Moosmann B. (2015). Protein-borne methionine residues as structural antioxidants in mitochondria. Amino Acids.

[2] Wolschner C., Giese A., Kretzschmar H.A., Huber R., Moroder L., Budisa N. (2009). Design of anti- and pro-aggregation variants to assess the effects of methionine oxidation in human prion protein. Proc. Natl. Acad. Sci. USA.

[3] Gilles A.M., Marlière P., Rose T., Sarfati R., Longin R., Meier A., Fermandjian S., Monnot M., Cohen G.N., Bârzu O. (1988). Conservative replacement of methionine by norleucine in *Escherichia coli* adenylate kinase. J. Biol. Chem..

[4] Cowie D.B., Cohen G.N. (1957). Biosynthesis by *Escherichia coli* of active altered proteins containing selenium instead of sulfur. Biochim. Biophys. Acta.

[5] Besse D., Budisa N., Karnbrock W., Minks C., Musiol H.J., Pegoraro S., Siedler F., Weyher E., Moroder L. (1997). Chalcogen-analogs of amino acids. Their use in X-ray crystallographic and folding studies of peptides and proteins. Biol. Chem..

[6] De Simone A., Acevedo-Rocha C.G., Hoesl M.G., Budisa N. (2016). Towards Reassignment of the Methionine Codon AUG to Two Different Noncanonical Amino Acids in Bacterial Translation. Croat. Chem. Acta.

[7] Yoshida A., Yamasaki M. (1959). Studies on the mechanism of protein synthesis; incorporation of ethionine into alpha-amylase of Bacillus subtilis. Biochim. Biophys. Acta.

[8] Tang Y., Tirrell D.A. (2002). Attenuation of the editing activity of the *Escherichia coli* leucyl-tRNA synthetase allows incorporation of novel amino acids into proteins in vivo. Biochemistry.

[9] Kiick K.L., Saxon E., Tirrell D.A., Bertozzi C.R. (2002). Incorporation of azides into recombinant proteins for chemoselective modification by the Staudinger ligation. Proc. Natl. Acad. Sci. USA.

[10] Bhushan B., Lin Y.A., Bak M., Phanumartwiwath A., Yang N., Bilyard M.K., Tanaka T., Hudson K.L., Lercher L., Stegmann M. (2018). Genetic Incorporation of Olefin Cross-Metathesis Reaction Tags for Protein Modification. J. Am. Chem. Soc..

[11] Truong F. (2013). Expanding Protein Sequence Space through Incorporation of Non-Canonical Amino Acids. Ph.D. Thesis.

[12] Law B.J.C., Struck A.-W., Bennett M.R., Wilkinson B., Micklefield J. (2015). Site-specific bioalkylation of rapamycin by the RapM 16-O-methyltransferase. Chem. Sci..

[13] Wang R., Zheng W., Luo M. (2014). A sensitive mass spectrum assay to characterize engineered methionine adenosyltransferases with S-alkyl methionine analogues as substrates. Anal. Biochem..

[14] Wang F., Singh S., Zhang J., Huber T.D., Helmich K.E., Sunkara M., Hurley K.A., Goff R.D., Bingman C.A., Morris A.J. (2014). Understanding molecular recognition of promiscuity of thermophilic methionine adenosyltransferase sMAT from Sulfolobus solfataricus. FEBS J..

[15] Singh S., Zhang J., Huber T.D., Sunkara M., Hurley K., Goff R.D., Wang G., Zhang W., Liu C., Rohr J. (2014). Facile chemoenzymatic strategies for the synthesis and utilization of S-adenosyl-L-methionine analogues. Angew. Chem. Int. Ed. Engl..

[16] Meinnel T., Mechulam Y., Blanquet S. (1993). Methionine as translation start signal: A review of the enzymes of the pathway in *Escherichia coli*. Biochimie.

[17] Hatono S., Jimenez A., Wargovich M.J. (1996). Chemopreventive effect of S-allylcysteine and its relationship to the detoxification enzyme glutathione S-transferase. Carcinogenesis.

[18] Borlinghaus J., Albrecht F., Gruhlke M., Nwachukwu I., Slusarenko A. (2014). Allicin: Chemistry and Biological Properties. Molecules.

[19] Weiss N., Ide N., Abahji T., Nill L., Keller C., Hoffmann U. (2006). Aged Garlic Extract Improves Homocysteine-Induced Endothelial Dysfunction in Macro- and Microcirculation. J. Nutr..

[20] Stevens C.M., Johnson C.A., Watanabe R. (1955). Preparation of S-allyl-DL-homocysteine and related compounds and tests of their growth effects in rats. J. Biol. Chem..

[21] Völler J.-S., Budisa N. (2017). Coupling genetic code expansion and metabolic engineering for synthetic cells. Curr. Opin. Biotechnol..

[22] Exner M.P., Kuenzl T., To T.M.T., Ouyang Z., Schwagerus S., Hoesl M.G., Hackenberger C.P.R., Lensen M.C., Panke S., Budisa N. (2017). Design of S-Allylcysteine in Situ Production and Incorporation Based on a Novel Pyrrolysyl-tRNA Synthetase Variant. ChemBioChem.

[23] Budisa N., Pipitone O., Siwanowicz I., Rubini M., Pal P., Holak T., Gelmi M. (2004). Efforts towards the Design of “Teflon” Proteins:In vivo Translation with Trifluorinated Leucine and Methionine Analogues. Chem. Biodivers..

[24] Yoo T.H., Tirrell D.A. (2007). High-Throughput Screening for Methionyl-tRNA Synthetases That Enable Residue-Specific Incorporation of Noncanonical Amino Acids into Recombinant Proteins in Bacterial Cells. Angew. Chem. Int. Ed. Engl..

[25] Montclare J.K., Tirrell D.A. (2006). Evolving Proteins of Novel Composition. Angew. Chem. Int. Ed. Engl..

[26] Yoo T.H., Link A.J., Tirrell D.A. (2007). Evolution of a fluorinated green fluorescent protein. Proc. Natl. Acad. Sci. USA.

[27] Nagasundarapandian S., Merkel L., Budisa N., Govindan R., Ayyadurai N., Sriram S., Yun H., Lee S.-G. (2010). Engineering Protein Sequence Composition for Folding Robustness Renders Efficient Noncanonical Amino acid Incorporations. ChemBioChem.

[28] Nischan N., Herce H.D., Natale F., Bohlke N., Budisa N., Cardoso M.C., Hackenberger C.P.R. (2015). Covalent attachment of cyclic TAT peptides to GFP results in protein delivery into live cells with immediate bioavailability. Angew. Chem. Int. Ed. Engl..

[29] Agostini F., Völler J.-S., Koksch B., Acevedo-Rocha C.G., Kubyshkin V., Budisa N. (2017). Biocatalysis with Unnatural Amino Acids: Enzymology Meets Xenobiology. Angew. Chem. Int. Ed. Engl..

[30] Ma Y. (2016). Metabolic Engineering of O-acetyl-L-homoserine Sulfhydrylase and Met-Biosynthetic Pathway in *Escherichia coli*. Ph.D. Thesis.

[31] Ma Y., Di Salvo M.L., Budisa N. (2018). Self-Directed in Cell Production of Methionine Analogue Azidohomoalanine by Synthetic Metabolism and Its Incorporation into Model Proteins. Methods. Mol. Biol..

[32] Di Salvo M.L., Budisa N., Contestabile R., Hicks M.G., Kettner C. PLP-dependent Enzymes: A Powerful Tool for Metabolic Synthesis of Non-canonical Amino Acids. Proceedings of the Beilstein Bozen Symposium on Molecular Engineering and Control.

[33] Budisa N. (2005). Engineering the Genetic Code.

[34] Ma Y., Biava H., Contestabile R., Budisa N., Di Salvo M. (2014). Coupling Bioorthogonal Chemistries with Artificial Metabolism: Intracellular Biosynthesis of Azidohomoalanine and Its Incorporation into Recombinant Proteins. Molecules.

[35] Ferla M.P., Patrick W.M. (2014). Bacterial methionine biosynthesis. Microbiology.

[36] Ma Y., Al-Shameri A., Schipp C.J., Contestabile R., Budisa N., Di Salvo M.L. (2019). Metabolically reconfigured *Escherichia coli* with self-directed in-cell production of synthetic amino acids as substrates for reprogrammed protein translation.

[37] Greene R.C., Neidhardt F. (1996). Biosynthesis of Methionine. Escherichia coli and Salmonella typhimurium, Cellular and Molecular Biology.

[38] Wiltschi B., Merkel L., Budisa N. (2009). Fine tuning the N-terminal residue excision with methionine analogues. ChemBioChem.

[39] Yesildag C., Ouyang Z., Zhang Z., Lensen M.C. (2019). Micro-Patterning of PEG-Based Hydrogels With Gold Nanoparticles Using a Reactive Micro-Contact-Printing Approach. Front. Chem..

[40] Torres-Kolbus J., Chou C., Liu J., Deiters A. (2014). Synthesis of Non-linear Protein Dimers through a Genetically Encoded Thiol-ene Reaction. PLoS ONE.

[41] Kharkar P.M., Rehmann M.S., Skeens K.M., Maverakis E., Kloxin A.M. (2016). Thiol-ene click hydrogels for therapeutic delivery. ACS Biomater. Sci. Eng..

[42] Isidro-Llobet A., Ivarez M., Alberiicio F. (2009). Amino acid-protecting groups. Chem. Rev..

[43] Li J., Yu J., Zhao J., Wang J., Zheng S., Lin S., Chen L., Yang M., Jia S., Zhang X. (2014). Palladium-triggered deprotection chemistry for protein activation in living cells. Nat. Chem..

[44] Heyrovský M., Vavřička S. (1999). Electrochemical reactivity of homocysteine at mercury electrodes as compared with cysteine. Bioelectrochem. Bioenerg..

[45] Kasper M.-A., Glanz M., Stengl A., Penkert M., Klenk S., Sauer T., Schumacher D., Helma J., Krause E., Cardoso M.C. (2019). Cysteine-selective phosphonamidate electrophiles for modular protein bioconjugations. Angew. Chem. Int. Ed. Engl..

[46] Siebertz K.D., Hackenberger C.P.R. (2018). Chemoselective triazole-phosphonamidate conjugates suitable for photorelease. Chem. Commun..

[47] Mark S.S. (2011). Bioconjugation Protocols.

[48] Schumacher D., Hackenberger C.P.R. (2014). More than add-on: Chemoselective reactions for the synthesis of functional peptides and proteins. Curr. Opin. Chem. Biol..

[49] Budisa N., Karnbrock W., Steinbacher S., Humm A., Prade L., Neuefeind T., Moroder L., Huber R. (1997). Bioincorporation of telluromethionine into proteins: A promising new approach for X-ray structure analysis of proteins. J. Mol. Biol..

[50] Liu C.C., Schultz P.G. (2010). Adding new chemistries to the genetic code. Annu. Rev. Biochem..

[51] McKay C.S., Finn M.G. (2014). Click Chemistry in Complex Mixtures: Bioorthogonal Bioconjugation. Chem. Biol..

[52] Hoyle C.E., Bowman C.N. (2010). Thiol-Ene Click Chemistry. Angew. Chem. Int. Ed. Engl..

[53] Exner M., Köhling S., Rivollier J., Gosling S., Srivastava P., Palyancheva Z., Herdewijn P., Heck M.-P., Rademann J., Budisa N. (2016). Incorporation of Amino Acids with Long-Chain Terminal Olefins into Proteins. Molecules.

[54] Teramoto H., Kojima K. (2015). Incorporation of Methionine Analogues Into Bombyx mori Silk Fibroin for Click Modifications. Macromol. Biosci..

[55] Zhang Q., Shi X., Jiang Y., Li Z. (2014). Influence of α-methylation in constructing stapled peptides with olefin metathesis. Tetrahedron.

[56] Köhling S., Exner M.P., Nojoumi S., Schiller J., Budisa N., Rademann J. (2016). One-Pot Synthesis of Unprotected Anomeric Glycosyl Thiols in Water for Glycan Ligation Reactions with Highly Functionalized Sugars. Angew. Chem. Int. Ed. Engl..

[57] Lowe A.B. (2010). Thiol-ene “click” reactions and recent applications in polymer and materials synthesis. Polym. Chem..

[58] Chen Y.X., Triola G., Waldmann H. (2011). Bioorthogonal chemistry for site-specific labeling and surface immobilization of proteins. Acc. Chem. Res..

[59] Buhl M., Vonhören B., Ravoo B.J. (2015). Immobilization of Enzymes via Microcontact Printing and Thiol–Ene Click Chemistry. Bioconjug. Chem..

[60] Fallacara A., Baldini E., Manfredini S., Vertuani S. (2018). Hyaluronic Acid in the Third Millennium. Polymers (Basel).

[61] Jakubowski H. (2004). Molecular basis of homocysteine toxicity in humans. Cell. Mol. Life Sci..

[62] Sekowska A., Dénervaud V., Ashida H., Michoud K., Haas D., Yokota A., Danchin A. (2004). Bacterial variations on the methionine salvage pathway. BMC Microbiol..

[63] Krömer J.O., Wittmann C., Schröder H., Heinzle E. (2006). Metabolic pathway analysis for rational design of L-methionine production by *Escherichia coli* and Corynebacterium glutamicum. Metab. Eng..

[64] Weissbach H., Brot N. (1991). Regulation of methionine synthesis in *Escherichia coli*. Mol. Microbiol..

[65] Su C.-H., Greene R.C. (1970). Regulation of Methionine Biosynthesis in *Escherichia coli*: Mapping of the metJ Locus and Properties of a metJ+/metJ- Diploid. Proc. Natl. Acad. Sci. USA.

[66] Huang J.-F., Liu Z.-Q., Jin L.-Q., Tang X.-L., Shen Z.-Y., Yin H.-H., Zheng Y.-G. (2017). Metabolic engineering of *Escherichia coli* for microbial production of L-methionine. Biotechnol. Bioeng..

[67] Li H., Wang B.S., Li Y.R., Zhang L., Ding Z.Y., Gu Z.H., Shi G.Y. (2017). Metabolic engineering of *Escherichia coli* W3110 for the production of l-methionine. J. Ind. Microbiol. Biotechnol..

[68] Nielsen J., Keasling J.D. (2016). Engineering Cellular Metabolism. Cell.

[69] Giese C., Lepthien S., Metzner L., Brandsch M., Budisa N., Lilie H. (2008). Intracellular uptake and inhibitory activity of aromatic fluorinated amino acids in human breast cancer cells. ChemMedChem.

[70] Acevedo-Rocha C.G., Geiermann A.-S., Budisa N., Merkel L. (2012). Design of protein congeners containing β-cyclopropylalanine. Mol. Biosyst..

[71] Hoesl M.G., Oehm S., Durkin P., Darmon E., Peil L., Aerni H.-R., Rappsilber J., Rinehart J., Leach D., Söll D. (2015). Chemical Evolution of a Bacterial Proteome. Angew. Chem. Int. Ed. Engl..

[72] Budisa N., Steipe B., Demange P., Eckerskorn C., Kellermann J., Huber R. (1995). High-level Biosynthetic Substitution of Methionine in Proteins by its Analogs 2-Aminohexanoic Acid, Selenomethionine, Telluromethionine and Ethionine in *Escherichia coli*. Eur. J. Biochem..

[73] Wiltschi B., Wenger W., Nehring S., Budisa N. (2008). Expanding the genetic code of Saccharomyces cerevisiae with methionine analogues. Yeast.

[74] Lepthien S., Merkel L., Budisa N. (2010). In vivo double and triple labeling of proteins using synthetic amino acids. Angew. Chem. Int. Ed. Engl..

[75] Bohlke N. (2014). Protein Engineering and Bioorthogonal Chemistry for Multivalent Scaffold Design Global Reassignment of Rare Codons in the Genetic Code of *Escherichia coli*. Ph.D. Thesis.

