# Supplementary material for: In-Cell Synthesis of Bioorthogonal Alkene Tag S-Allyl-Homocysteine and Its Coupling with Reprogrammed Translation
Source: Int J Mol Sci. 2019 May 9;20(9):2299. doi: 10.3390/ijms20092299 (PMC6539321; doi:10.3390/ijms20092299)
Supplement: Supplementary file 1 [file ijms-20-02299-s001.pdf]

In-cell synthesis of bioorthogonal alkene tag S-allyl-homocysteine and its coupling with reprogrammed translation – Supplementary Information

Saba Nojoumi[1], Ying Ma[1], Sergej Schwagerus[2,3], Christian P. R. Hackenberger[2,3], Nediljko Budisa[1,4*]

1 M. Sc. Saba Nojoumi, Dr. Ying-Katrina Ma, Prof. Dr. Nediljko Budisa; Institut für Chemie, Technische Universität Berlin; Müller-Breslau-Str. 10, D-10623 Berlin; E-Mail: nediljko.budisa@tu-berlin.de

2 M. Sc. Sergej Schwagerus, Prof. Dr. Christian P. R. Hackenberger; Institut für Chemie der Humboldt-Universität zu Berlin, Brook-Taylor-Str. 2, D-12489 Berlin; hackenbe@fmp-berlin.de

3 Leibniz-Forschungsinstitut für Molekulare Pharmakologie (FMP), Campus Berlin-Buch, Robert-Roessle-Str. 10, D-13125 Berlin, Germany; E-Mail: hackenbe@fmp-berlin.de

4 Chair of Chemical Synthetic Biology, Department of Chemistry, University of Manitoba, 144 Dysart Rd, R3T 2N2 Winnipeg, MB, Canada, E-Mail: nediljko.budisa@umanitoba.ca

* Correspondence: nediljko.budisa@tu-berlin.de; nediljko.budisa@umanitoba.ca
Tel.: +49303142882 / +12044749178

1. Protein design and analytics

Protein construct variants were designed *in silico* with CAD program PyMol (The PyMOL Molecular Graphics System, Version 2.0 Schrödinger, LLC) and DNA vectors were cloned *in silico* with Geneious (Geneious 7.1.7 (https://www.geneious.com)).

1.1 *cfGFPhs1-RM(1Sahc) (x1NSahc)* ***10*** (mutant with N-terminal Met only)

1.1.1. Protein sequence information

- C-terminal (His)$_6$-tag removed by TEV protease
- Sahc at position 1 of N-terminus with Q as the penultimate residue.

```
        10         20         30         40         50         60
MQSKGEELFT GVVPILVELD GDVNGHKFSV RGEGEGDATN GKLTLKFIST TGKLPVPWPT
        70         80         90        100        110        120
LVTTLGYGVQ SFARYPDHIK RHDFFKSALP EGYVQERTIS FKDDGTYKTR AEVKFEGDTL
       130        140        150        160        170        180
VNRIELKGID FKEDGNILGH KLEYNFNSHK VYITADKQKN GIKANFKIRH NVEDGSVQLA
       190        200        210        220        230        240
DHYQQNTPIG DGPVLLPDNH YLSTQSVLLK DPNEKRDHAV LLEFVTAAGI THGKDELYKE
NLYFQ
```

1.1.2. Mass analyses:

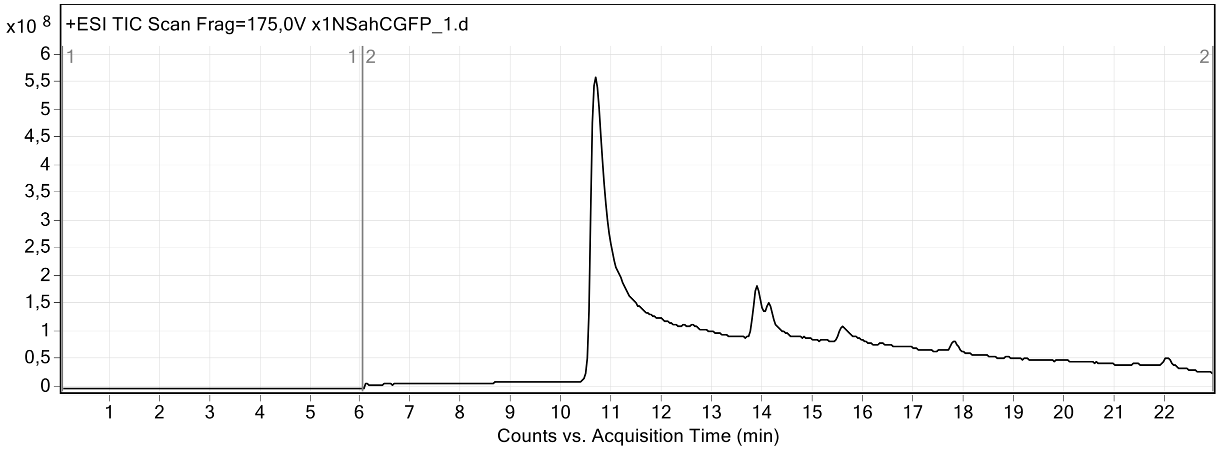

Figure S1. TIC (total ion count) scan of HPLC run for cfGFPhs1-RM(1Sahc). Distinct curve detected t_R = 10.455-12.272 min.

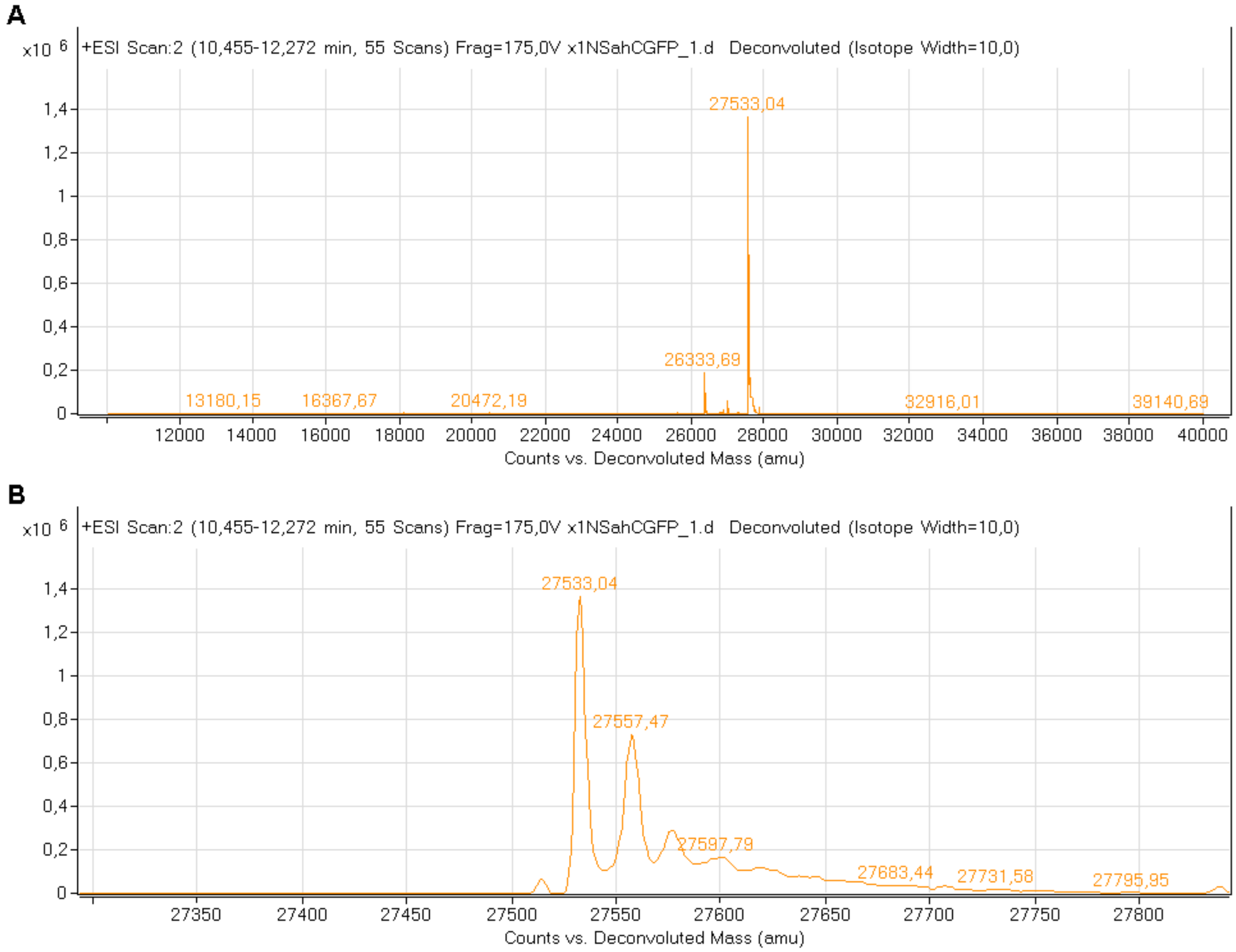

Figure S2. Deconvoluted full spectrum of cfGFPhs1-RM(134Sahc). (**A**) Total range: 10 kDa – 40 kDa with (**B**) detailed view. Assignment of mass peaks: (i) 27533.04 Da – detected MW cfGFPhs1-RM(1Met): calculated MW: 27532.99 Da. (ii) 27557.51 Da – detected MW of fully labelled cfGFPhs1-RM(1Sahc): expected/calculated MW: 27558.99 Da. All further peaks are undefined Na^+ adducts.

Assignment of mass peaks:

m/z (Da)	Height	Start X	End X	Area	Ion species
27533.04	1362446.41	27525	27542	8984176	cfGFPhs1-RM(1Met) $[M+H]^+$
**27557.47**	**729132.06**	**27544**	**27567**	**7775432**	**cfGFPhs1-RM(1Sahc)**
27576.80	292249.41	27567	27588	4278068	undefined adduct

1.1.3. Protein yield and Met to Sahc substitution level

x1NSahc with 1 Sahc at position M1 without histidine tag: 2.0 ml, 0.2 mg mL^{-1} (50 mM NaH_2PO_4, 300 mM NaCl, 20% glycerol). Incorporation estimation by integration of mass curve: 57%.

1.1.4. SDS-PAGE analysis

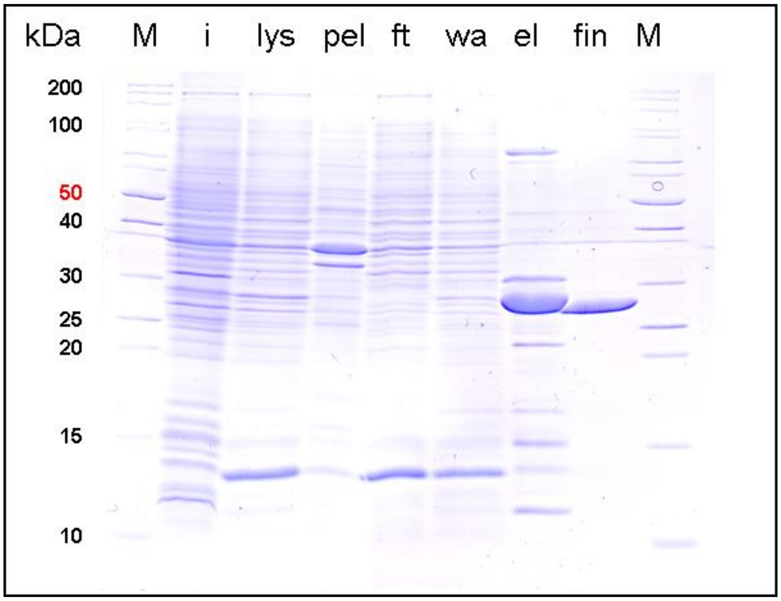

Figure S3. SDS-PAGE analysis gel of all stages of expression and purification cfGFPhs1-RM(1Sahc). M: Marker Prestained Protein Ladder, Thermo Scientific™; ni: non induced sample before expression; i: induced sample after expression; lys: lysate (soluble); pel: pellet (insoluble); ft: flow through; wa: first wash with N_B Ni-NTA column; el: eluate Ni-NTA column; fin: final protein fraction used for analyses and reactions.

1.2. cfGFPhs1-RM(134Sahc) (cfG1Sahc) **5** (mutant with single Met at internal position 134 after protein purification)

1.2.1. Protein sequence information

- N-terminal $(His)_6$-tag along with Met 1 removed by TEV protease cleavage
- Single methionine at position D134M

```
        10         20         30         40         50         60
SASKGEELFT GVVPILVELD GDVNGHKFSV RGEGEGDATN GKLTLKFIST TGKLPVPWPT
        70         80         90        100        110        120
LVTTLGYGVQ SFARYPDHIK RHDFFKSALP EGYVQERTIS FKDDGTYKTR AEVKFEGDTL
       130        140        150        160        170        180
VNRIELKGID FKEMGNILGH KLEYNFNSHK VYITADKQKN GIKANFKIRH NVEDGSVQLA
       190        200        210        220        230
DHYQQNTPIG DGPVLLPDNH YLSTQSVLLK DPNEKRDHAV LLEFVTAAGI THGKDELYK
```

1.2.2. Mass analyses

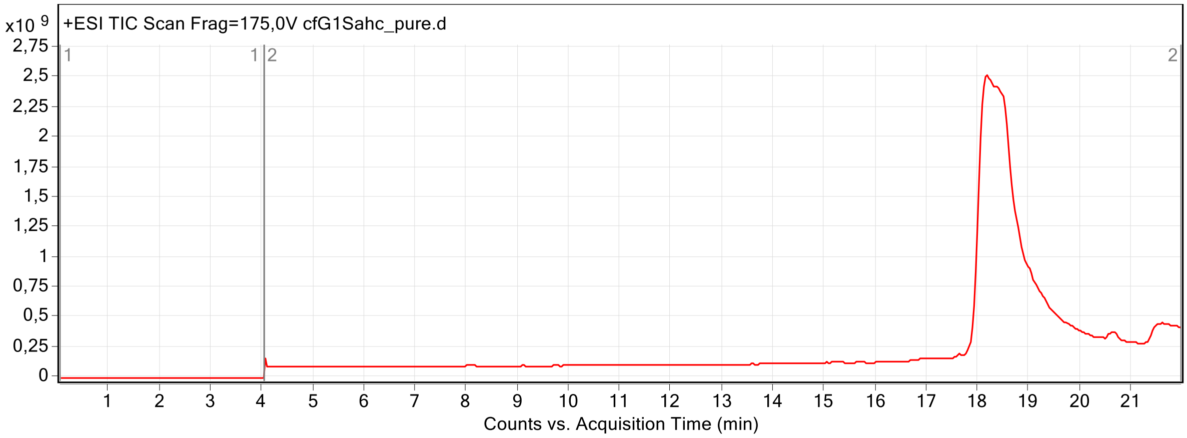

Figure S4. TIC (total ion count) scan of HPLC run for cfGFPhs1-RM(134Sahc). Distinct curve detected t_R = 17.939-19.452 min.

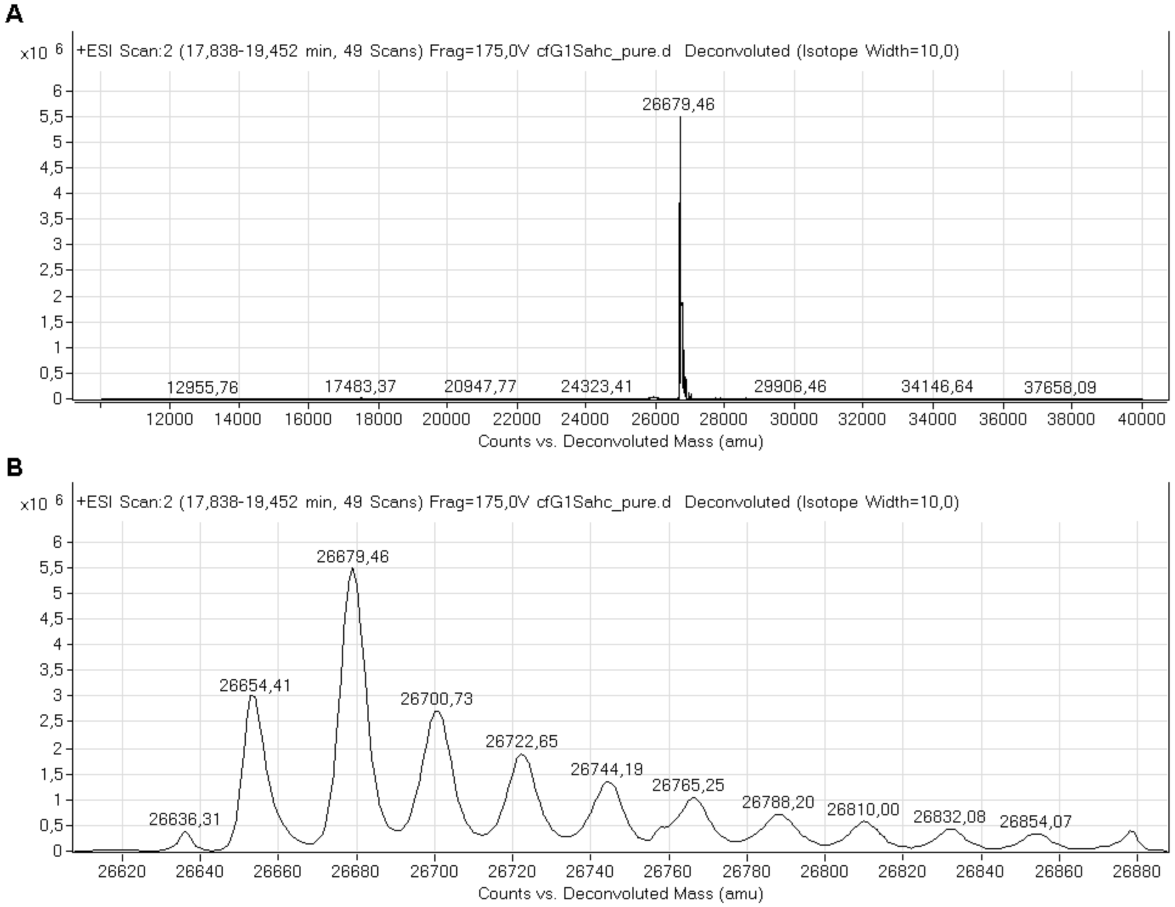

Figure S5. Deconvoluted full spectrum of cfGFPhs1-RM(134Sahc). (**A**) Total range 10 kDa - 40 kDa with (**B**) detailed view. Assignment of mass peaks: (i) 27554.41 Da – detected MW (cfGFPhs1-RM(134Met)), calculated MW: 27554 Da; (ii) 26679.46 Da – detected MW (cfGFPhs1-RM(134Sahc)), calculated MW: 26679.0 Da; (ii) 26700.73 Da – detected MW of fully labelled (cfGFPhs1-RM(134Sahc) $[M+Na]^+$), calculated MW: 26701 Da; (iv) 26722.65 Da – detected MW (cfGFPhs1-RM(134Sahc) $[M+2Na]^+$), calculated MW: 26725 Da. All further signals belong to Na^+ adducts of fully labelled cfG1Sahc.

Assignment of mass peaks:

m/z (Da)	Height	Start X	End X	Area	Ion species
26654.41	2999397.48	26644	26664	23328049	cfGFPhs1-RM(134M)
**26679.46**	**5509124.98**	**26669**	**26689**	**45326232**	**cfGFPhs1-RM(134Sahc)**
26700.73	2726846.87	26690	26712	28201471	cfGFPhs1-RM(134M) $[M+2Na]^+$
26722.65	1899105.47	26712	26734	20083523	cfGFPhs1-RM(134Sahc) $[M+2Na]^+$
26744.19	1357273.6	26734	26754	14470452	cfGFPhs1-RM(134Sahc) $[M+3Na]^+$
26765.25	1049067.75	26754	26777	12887384	cfGFPhs1-RM(134Sahc) $[M+4Na]^+$
26788.20	721758.46	26777	26800	9043953	cfGFPhs1-RM(134Sahc) $[M+5Na]^+$
26810.00	578425.41	26800	26821	6234573	cfGFPhs1-RM(134Sahc) $[M+6Na]^+$
26832.08	447515.78	26821	26843	4610025	cfGFPhs1-RM(134Sahc) $[M+7Na]^+$
26854.07	344186.44	26843	26866	3812381	cfGFPhs1-RM(134Sahc) $[M+8Na]^+$

1.2.3. Protein yield and Met to Sahc substitution level

cfGFPhs1-RM(134Sahc) with 1 Sahc at position D134M without histidine tag: 5.0 ml, 9.20 mg mL^{-1} (50 mM NaH_2PO_4, 300 mM NaCl, 20% glycerol). Incorporation estimation by integrated mass curve: 86%.

1.2.4. SDS-PAGE analysis

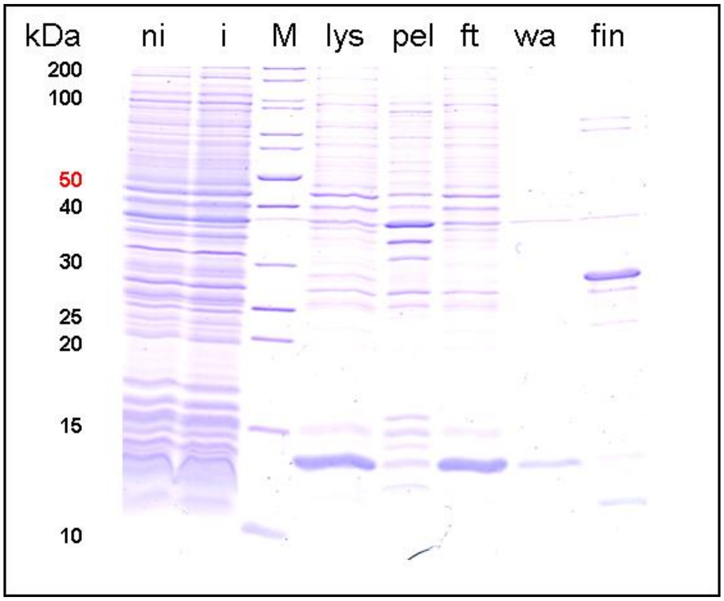

Figure S6. SDS-PAGE analysis gel of all stages of expression and purification of cfGFPhs1-RM(134Sahc). M: Marker Prestained Protein Ladder, Thermo Scientific™; ni: non induced sample before expression; i: induced sample after expression; lys: lysate (soluble); pel: pellet (insoluble); ft: flow through; wa: first wash with N_B Ni-NTA column; fin: final protein fraction used for analyses and reactions.

1.3. *cfGFPhs1-RM(134Sahc:143Sahc) (cfG2Sahc)* **6** (mutant with two Met residues at internal postions 134 and 143 after protein purification)

1.3.1. Protein sequence information

- N-terminal $(His)_6$-tag along with Met1 removed by TEV protease
- Sahc at positions D134M und E143M (two in-frame Met residues)

```
        10         20         30         40         50         60
SASKGEELFT GVVPILVELD GDVNGHKFSV RGEGEGDATN GKLTLKFIST TGKLPVPWPT
        70         80         90        100        110        120
LVTTLGYGVQ SFARYPDHIK RHDFFKSALP EGYVQERTIS FKDDGTYKTR AEVKFEGDTL
       130        140        150        160        170        180
VNRIELKGID FKEMGNILGH KLMYNFNSHK VYITADKQKN GIKANFKIRH NVEDGSVQLA
       190        200        210        220        230
DHYQQNTPIG DGPVLLPDNH YLSTQSVLLK DPNEKRDHAV LLEFVTAAGI THGKDELYK
```

1.3.2. Mass analysis

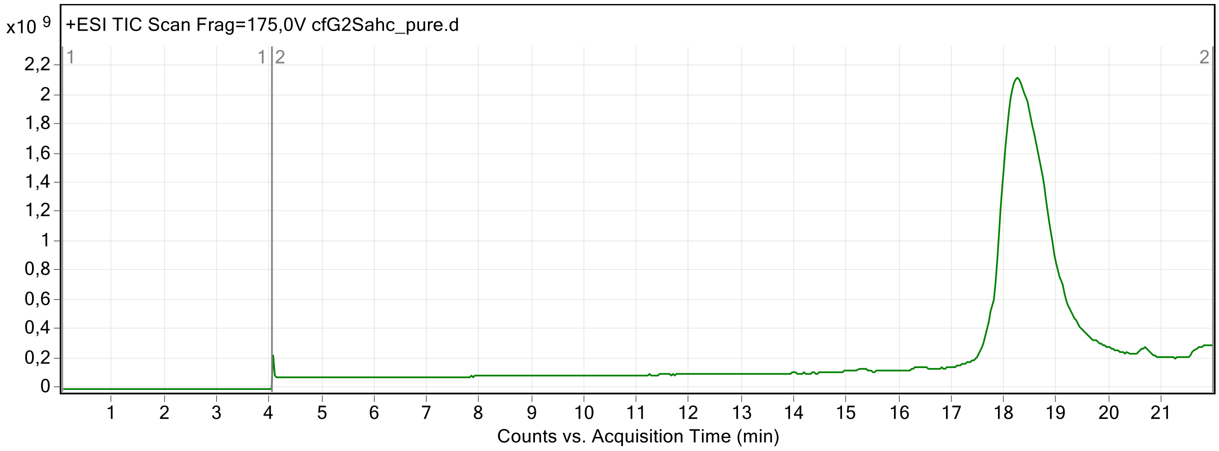

Figure S7. TIC (total ion count) scan of HPLC run for cfGFPhs1-RM(134Sahc:143Sahc). Distinct curve detected t_R = 17.504-19.590 min.

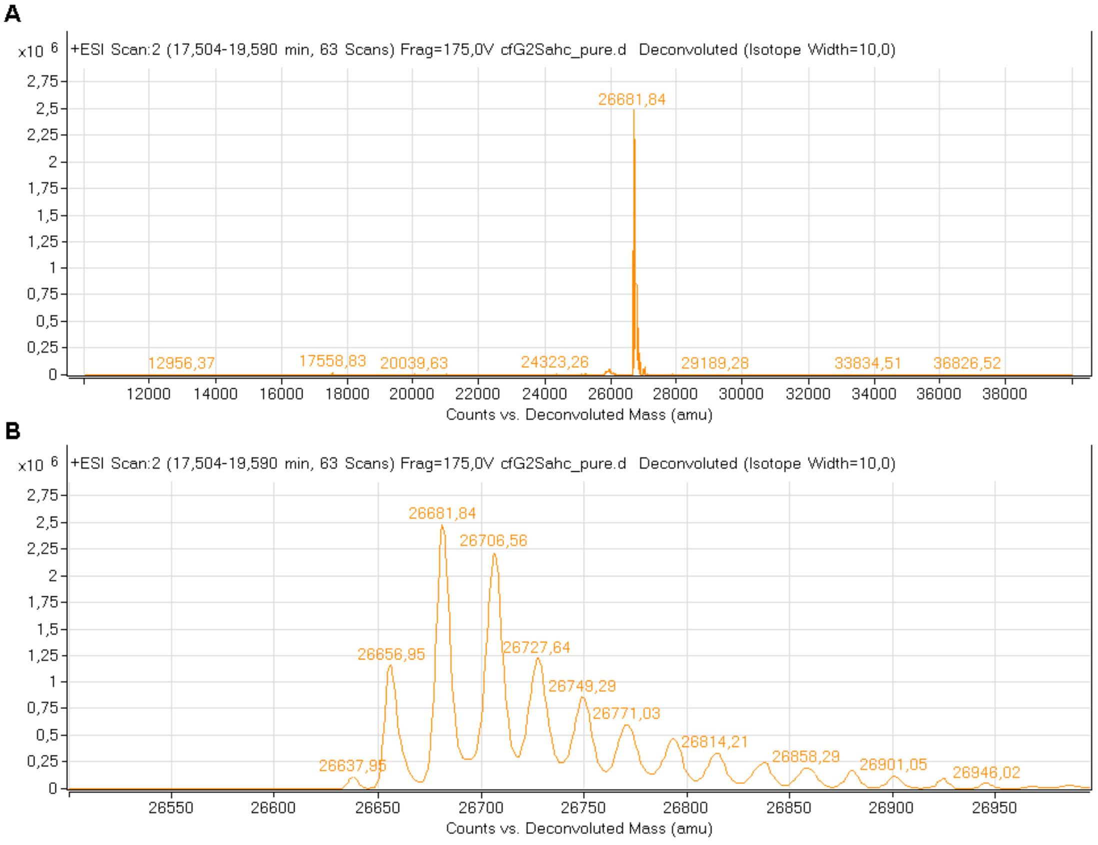

Figure S8. Deconvoluted full spectrum of cfGFPhs1-RM (134Sahc:143Sahc). (**A**) Total range 10 kDa - 40 kDa with (**B**) detailed view. Assignment of mass peaks: (i) 26656.95 Da – detected MW (cfGFPhs1-RM(134Met:143Met)), calculated MW: 26655 Da; (ii) 26681.84 Da – detected MW (cfGFPhs1-RM(134Sahc:143Met)), calculated MW: 26682.14 Da; (iii) 26706.56 Da – detected MW (cfGFPhs1-RM(134Sahc:143Sahc)), calculated MW: 26707.14 Da; (iv) 26727.64 Da – detected MW (cfGFPhs1-RM(134Sahc:143Sahc) $[M+Na]^+$), calculated MW: 26729 Da. All further signals belong to Na^+ adducts of fully labelled cfG2Sahc.

Assignment of mass peaks:

m/z (Da)	Height	Start X	End X	Area	Ion species
26656.95	1166721.55	26646	26666	9380648	cfGFPhs1-RM (134M:143M)
26681.84	2479611.68	26671	26693	21424578	cfGFPhs1-RM (134Sahc:143M)
**26706.56**	**2223765.04**	**26693**	**26717**	**24000240**	**cfGFPhs1-RM (134Sahc:143Sahc)**
26727.64	1224950.78	26717	26739	14896270	cfGFPhs1-RM (134Sahc:143Sahc) $[M+Na]^+$
26749.29	863435.95	26739	26760	10292107	cfGFPhs1-RM (134Sahc:143Sahc) $[M+2Na]^+$
26771.03	606685.26	26760	26783	7991486	cfGFPhs1-RM (134Sahc:143Sahc) $[M+3Na]^+$
26793.04	466490.34	26783	26804	5702461	cfGFPhs1-RM (134Sahc:143Sahc) $[M+4Na]^+$
26814.21	340818.32	26804	26826	3998993	cfGFPhs1-RM (134Sahc:143Sahc) $[M+5Na]^+$
26836.59	245345.35	26826	26848	2986485	cfGFPhs1-RM (134Sahc:143Sahc) $[M+6Na]^+$
26858.29	198927.26	26848	26870	2340530	cfGFPhs1-RM (134Sahc:143Sahc) $[M+7Na]^+$
26880.22	184798.89	26870	26890	1502406	cfGFPhs1-RM (134Sahc:143Sahc) $[M+8Na]^+$
26901.05	124635.95	26891	26911	1118862	cfGFPhs1-RM (134Sahc:143Sahc) $[M+9Na]^+$
26924.14	98265.85	26914	26934	709775	cfGFPhs1-RM (134Sahc:143Sahc) $[M+10Na]^+$
26946.02	55041.08	26938	26957	424051	cfGFPhs1-RM (134Sahc:143Sahc) $[M+11Na]^+$

1.3.3. Protein yield and Met to Sahc substitution level

cfG2Sahc with 2 Sahc at positions D134M and E143M without histidine tag: 5.5 ml, 10.1 mg mL^{-1} (50 mM NaH_2PO_4, 300 mM NaCl, 20% glycerol). Estimation of overall Met to Sahc substitution by integration of peaks: 71%. It should be noted that it is difficult to accurately assess the occupancy (i.e., the degree of Met-to-Sahc replacement) for each particular side chain because the SPI as substitution method works in a statistical fashion.

1.3.4. SDS-PAGE

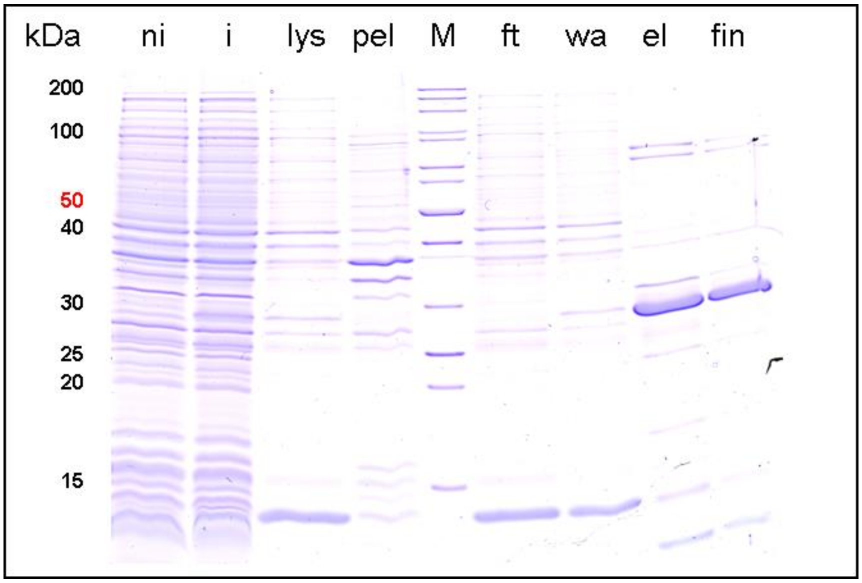

Figure S9. SDS-PAGE analysis gel of all stages of expression and purification cfGFPhs1-RM (134Sahc:143Sahc). M: Marker Prestained Protein Ladder, Thermo Scientific™; ni: non induced sample before expression; i: induced sample after expression; lys: lysate (soluble); pel: pellet (insoluble); ft: flow through; wa: first wash with N_B Ni-NTA column; el: eluate Ni-NTA column; fin: final protein fraction used for analyses and reactions.

1.4. *cfGFPhs1-RM(1Sahc:134Sahc:143Sahc)* – (mutant with N-terminal Met and two Met residues at the internal positions in protein sequence (“triple Met-mutant”))

1.4.1. Protein sequence information

- C-terminal (His)₆-tag
- Sahc at positions M1, D134M, E143M (with Q as the penultimate residue.)

```
        10         20         30         40         50         60
MQSKGEELFT GVVPILVELD GDVNGHKFSV RGEGEGDATN GKLTLKFIST TGKLPVPWPT
        70         80         90        100        110        120
LVTTLGYGVQ SFARYPDHIK RHDFFKSALP EGYVQERTIS FKDDGTYKTR AEVKFEGDTL
       130        140        150        160        170        180
VNRIELKGID FKEMGNILGH KLMYNFNSHK VYITADKQKN GIKANFKIRH NVEDGSVQLA
       190        200        210        220        230        240
DHYQQNTPIG DGPVLLPDNH YLSTQSVLLK DPNEKRDHAV LLEFVTAAGI THGKDELYKE
       250
NLYFQSHHHH HH
```

1.4.2. Mass analysis

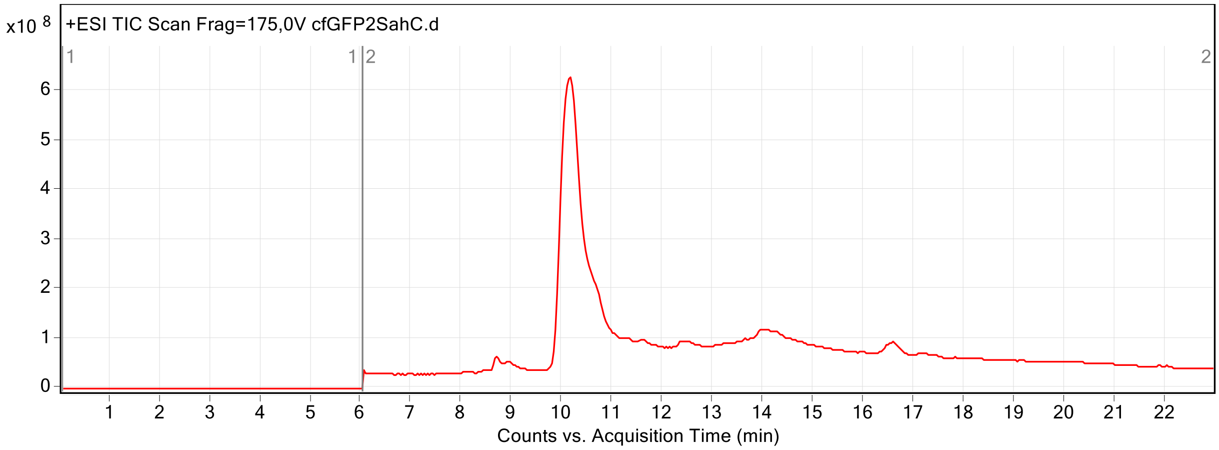

Figure S10. TIC (total ion count) scan of HPLC run for cfGFPhs1-RM(1Sahc:134Sahc:143Sahc). Distinct curve detected t_R = 9.855-11.272 min.

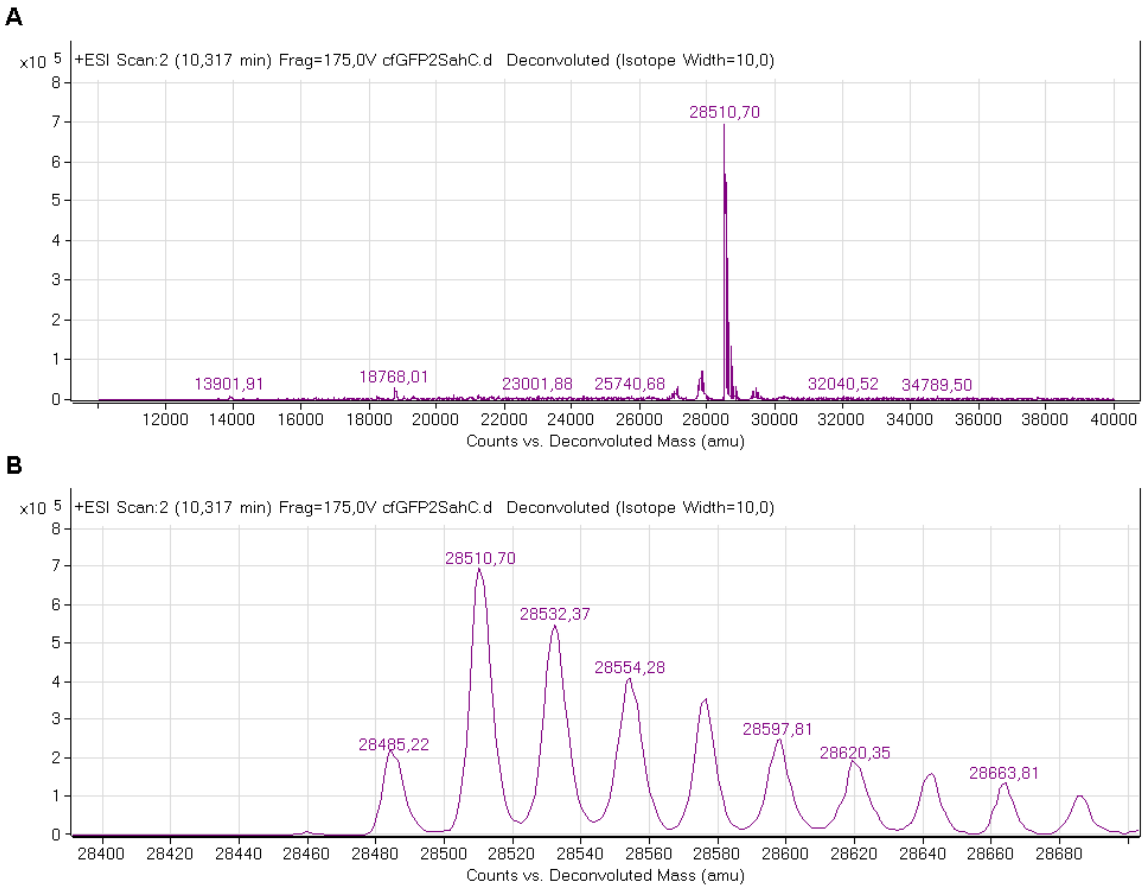

Figure S11. Deconvoluted full spectrum of cfGFPhs1-RM(1Sahc:134Sahc:143Sahc). (**A**) total range 10 kDa - 40 kDa with (**B**) detailed view. Assignment of mass peaks: (i) 28485.22 Da – detected MW (cfGFPhs1-RM(1Met:134Sahc:143Met)): calculated MW: 28487 Da; (ii) 28510.70 Da – detected MW (cfGFPhs1-RM(1Met:134Sahc:143Sahc)): calculated MW: 28513.14 Da; (iii) 28532.37 Da – detected MW (cfGFPhs1-RM(1Sahc:134Sahc:143Sahc)): calculated MW: 28539.14 Da; (iv) 28554.28 Da - detected MW (cfGFPhs1-RM(1Sahc:134Sahc:143Sahc) $[M+Na]^+$): calculated MW: 28555.14 Da. All further signals belong to Na^+ adducts of cfG3Sahc.

Assignment of mass peaks:

m/z (Da)	Height	Start X	End X	Area	Ion species
28485.22	206831.30	28477	28497	1678975	cfGFPhs1-RM(1M:134Sahc:143M)
28510.70	694426.19	28500	28522	5539262	cfGFPhs1-RM(1M:134Sahc:143Sahc)
**28532.37**	**547167.00**	**28522**	**28542**	**4671782**	**cfGFPhs1-RM(1Sahc:134Sahc:143Sahc)**
28554.28	409072.09	28544	28566	3701978	cfGFPhs1-RM(1Sahc:134Sahc:143Sahc) $[M+Na]^+$
28576.12	356120.43	28566	28586	2766562	cfGFPhs1-RM(1Sahc:134Sahc:143Sahc) $[M+2Na]^+$
28597.81	246861.26	28587	28609	2109530	cfGFPhs1-RM(1Sahc:134Sahc:143Sahc) $[M+3Na]^+$
28620.35	186054.24	28610	28632	1751258	cfGFPhs1-RM(1Sahc:134Sahc:143Sahc) $[M+4Na]^+$
28641.87	159403.20	28632	28652	1287824	cfGFPhs1-RM(1Sahc:134Sahc:143Sahc) $[M+5Na]^+$
28663.81	134322.52	28656	28673	933412	cfGFPhs1-RM(1Sahc:134Sahc:143Sahc) $[M+6Na]^+$
28685.88	101479.46	28676	28696	793921	cfGFPhs1-RM(1Sahc:134Sahc:143Sahc) $[M+7Na]^+$

1.4.3. Protein yield and Met to Sahc substitution level

cfG3Sahc with 3 Sahc at positions M1, D134M, E143M with histidine tag: 2 ml, 0.66 mg mL^{-1} (50 mM NaH2PO4, 300 mM NaCl, 20% glycerol). Estimation of overall Met to Sahc substitution by integration of peaks: 71%. Here is also the difficulty to note to accurately assess the occupancy (i.e., the degree of Met-to-Sahc replacement) for each particular side chain because the SPI as substitution method works in a statistical fashion.

1.4.4. SDS-PAGE-gel

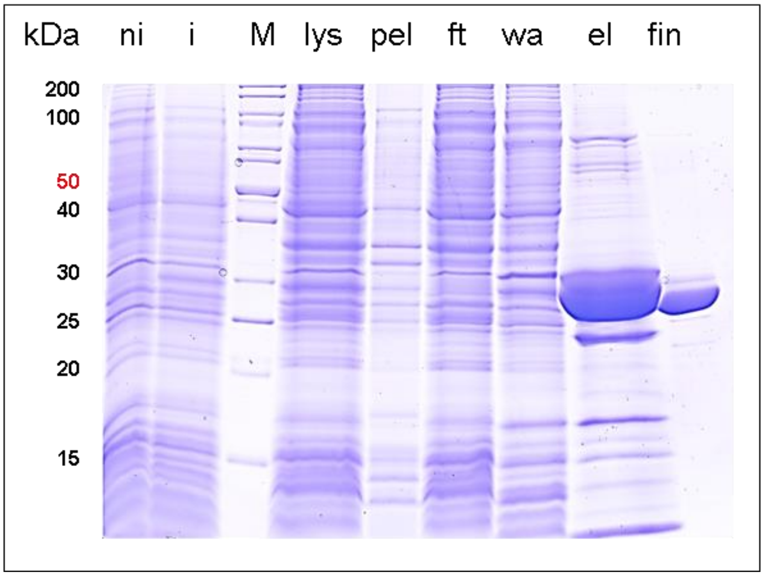

Figure S12. SDS-PAGE analysis gel of all stages of expression and purification of cfGFPhs1-RM(1Sahc:134Sahc:143Sahc). M: Marker Prestained Protein Ladder, Thermo Scientific™; ni: non induced sample before expression; i: induced sample after expression; lys: lysate (soluble); pel: pellet (insoluble); ft: flow through; wa: first wash with N_B Ni-NTA column; el: eluate Ni-NTA column; fin: final protein fraction used for analyses and reactions.

2. Syntheses of small ligands and precursors

2.1. 2-Acetamido-2-deoxy-β-D-galactopyranosyl-1-thiol (GalNAc) ***7****:*

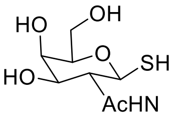

7

Figure S13. Structure of GalNAc **7**.

Synthesis by Sebastian Köhling [1].

2.2. O-acetyl-l-homocysteine (Oahc) **2***:*

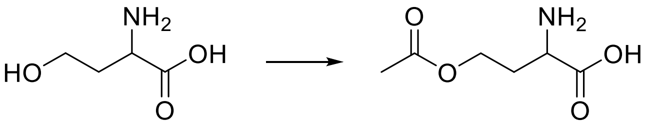

1 **2**

Figure S14. One-step reaction of L-homoserine **1** to Oahc **2**.

To a round bottomed flask containing a mixture of glacial acetic acid (99.9%, 17.8 mL) and perchloric acid (70%, 927 µL, 10.75 mmol, 1.28 eq.), was added the L-homoserine (1.0 g, 8.39 mmol, 1.0 eq.). While cooling the mixture to 17 °C, acetic anhydride (3.53 mL, 37.37 mmol, 4.45 eq.) was added carefully under stirring. The stirring was ceased for 90 min at room temperature. The reaction was then quenched by adding water (700 µL, 40.00 mmol) and stirred for another hour. Unreacted perchloric acid was decomposed by adding amyl amine (1.5 mL, 12.90 mmol). To the resulting mixture was added diethyl ether (200 mL) and kept at 4 °C overnight. The resulting precipitate was filtered off and the crude product (1.65 g) was dissolved in water (10 mL) and ethanol (70 mL). After standing overnight at 4°C a second precipitate was obtained. This product was isolated by filtration and yielded 554 mg (3.44 mmol, 41%) of the desired O-acetyl-L-homoserine **2** as a white solid.

ESI-MS: *m*/*z* = 162.0756 $[M + H]^+$; calculated for $[C_6H_{11}NO_4 + H]^+$: 162.0761.

^1H NMR (500 MHz, 10% CD_3OD in D_2O) δ: 4.25 (2H, t, *J* = 6.0 Hz, 4-H), 3.82 (1H, dd, *J* = 7.2, 5.3 Hz, 2-H), 2.26 - 2.32 (1H, m, 3-Ha), 2.16 - 2.23 (1H, m, 3-Hb), 2.11 (3H, s, 6-H).
^{13}C NMR (126 MHz, 10% CD_3OD in D_2O) δ: 175.1 (5-C), 174.7 (1-C), 62.7 (4-C), 53.8 (2-C), 30.4 (3-C), 21.4 (6-C).

2.3. Ethyl-N-phenyl-P-ethynyl phosphonamidate **15**

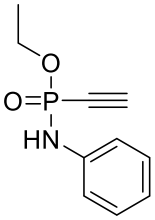

15

Figure S15. Structure of phenyl phosphonamidate (PP) **15**.

The compound **15** was synthesised according to the general procedure from 1.45 ml diethyl chlorophosphite (10.07 mmol) and 1.00 g phenyl azide (8.39 mmol). The crude phosphonamidate was

purified by flash column chromatography on silica gel (50% n-hexane in EtOAc) and obtained as a yellowish solid. (1.4 g, 6.74 mmol, 80.3%) [2].

^{1}H NMR (600 MHz, Chloroform-*d*) δ = 7.33 – 7.25 (m, 2H), 7.20 (d, *J*=7.6, 1H), 7.16 – 7.10 (m, 2H), 7.05 – 6.94 (m, 1H), 4.35 – 4.10 (m, 2H), 2.91 (d, *J*=12.9, 1H), 1.39 (t, *J*=7.1, 3H).
^{13}C NMR (151 MHz, Chloroform-*d*) δ = 139.18, 129.28, 122.23, 118.16 (d, *J*=7.6), 87.77 (d, *J*=48.8), 76.39 (d, *J*=272.9), 62.13 (d, *J*=5.1), 16.17 (d, *J*=7.4).
^{31}P NMR (243 MHz, Chloroform-*d*) δ=-8.75.HR-MS for $C_{10}H_{13}NO_2P^+$[M+H]$^+$ calcd.: 210.0678, found: 210.0680.

2.4. Generation of Pd(TPPTS)₄

3,3′,3″-Phosphanetriyltris(benzenesulfonic acid) trisodium salt (TPPTS) (6 eq., 15.2 mg, 26.8 µmol) was dissolved in 250 µl of PBS pH 7.4. Palladium(II) acetate (1.0 mg, 4.45 µmol) was added to generate the pale brown 17.8 mM $Pd(TPPTS)_4$.

2.5. Deallylation reactions of cfG1Sahc ***5*** *with Pd(TPPTS) catalyst*

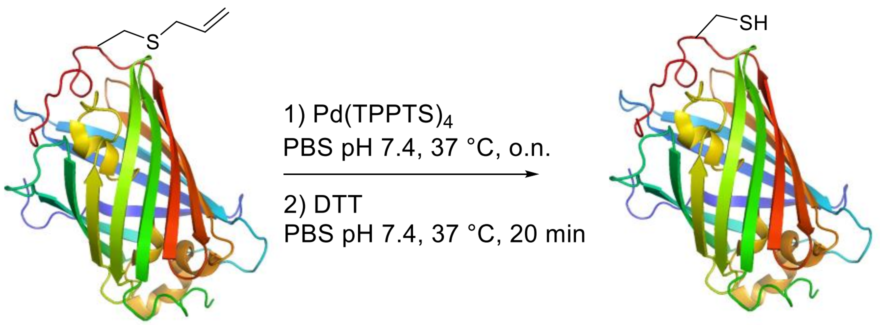

5

12

Figure S16. Deallylation reaction of cfGFPhs1-RM(134Sahc). cfG1Sahc **5** allowed to react to cfGFPhs1-RM(hC) **12**.

To cfG1Sahc **5** (1.78 nmol) in 36.3 µl PBS pH 7.4 was added the previous solution of $Pd(TPPTS)_4$ (17.8 mM). The deprotection was carried out with either 10 eq. (17.8 nmol, 0.1 µl) or 100 eq. (178 nmol, 1 µl) of $Pd(TPPTS)_4$. The mixtures were shaken at 37 °C overnight. Next a large excess of Dithiothreitol (DTT) (1000 eq., 1.78 µmol, 274 µg) was added and the solution was kept at 37 °C for 20 min, w/up. Full deprotection could be achieved with 100 eq. $Pd(TPPTS)_4$. The product cfGFPhs1-RM(hC) **12** was analysed by ESI QToF MS. Calculated MW: 26638 Da Detected MW: 26638 Da.

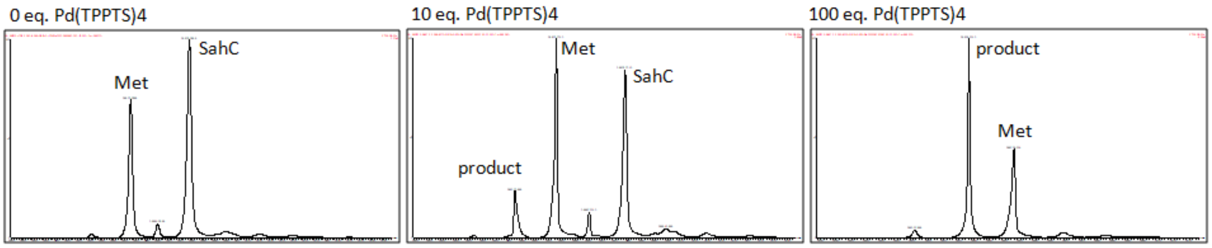

Figure S17. Deprotection progression with 10 and 100 eq. of the catalyst. Detected mass (ESI QToF) of starting material cfG1Sahc **5**: 26678 Da and the detected mass of the product **12**: 26638 Da.

2.6. Deallylation reactions of cfG2Sahc ***6*** *with Pd(TPPTS) catalyst*

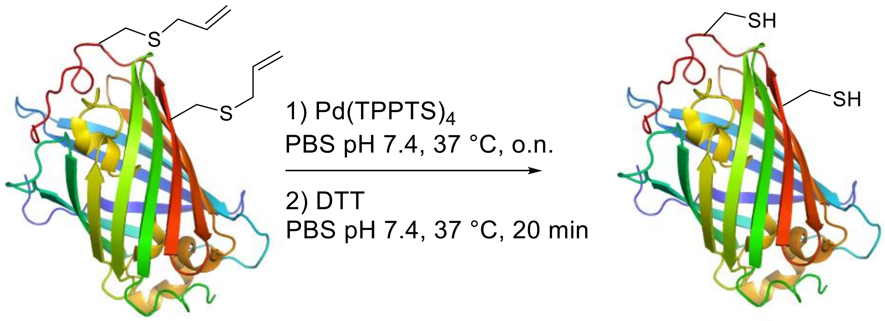

6

13

Figure 18. Deallylation reaction of cfGFPhs1-RM(134Sahc:143Sahc). cfG2Sahc **6** was allowed to react to cfGFPhs1-RM(2hc) **13**.

To cfG2Sahc **6** (1.78 nmol) in 36.3 µl PBS pH 7.4 was added the previous solution of Pd(TPPTS)₄ (17.8 mM). The deprotection was carried out with either 10 eq. (17.8 nmol, 0.1 µl) or 100 eq. (178 nmol, 1 µl) of Pd(TPPTS)₄. The mixtures were shaken at 37 °C overnight. Next a large excess of Dithiothreitol (DTT) (1000 eq., 1.78 µmol, 274 µg) was added and the solution was kept at 37 °C for 20 min, w/up. Full deprotection could be achieved with 100 eq. Pd(TPPTS)₄. The product cfGFPhs1-RM(2hC) **13** was analysed by ESI QToF MS. Calculated MW: 26627 Da; Detected MW: 26626 Da.

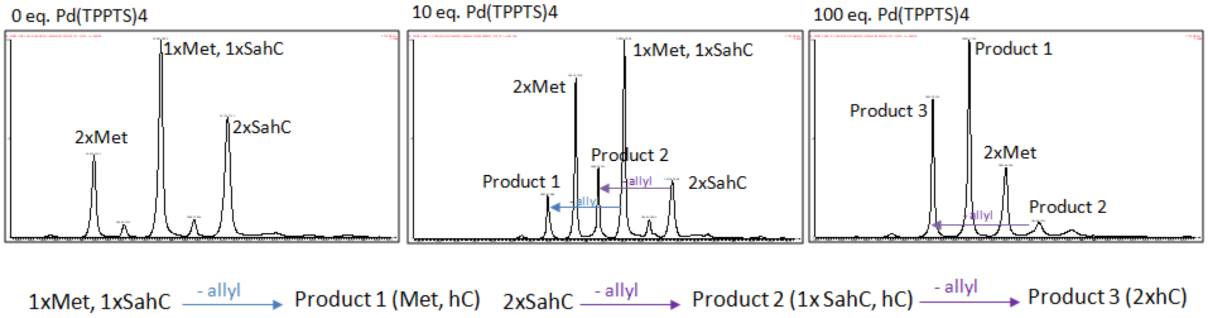

Figure S19. Deprotection progression with 10 and 100 eq. of the catalyst. Detected mass (ESI QToF) of starting material cfG2Sahc **6**: 26707 Da and the detected mass of the product **13**: 26626 Da.

*2.7. Conjugation of FAM maleimide **14** to fcGFPhs1-RM(hC) **12***

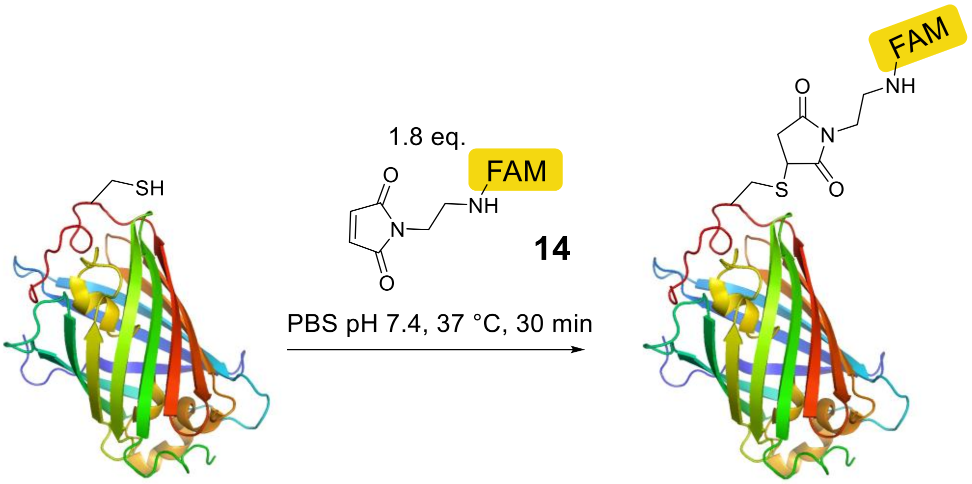

12 **16**

Figure S20. Conjugation reaction of fcGFPhs1-RM(hC) with FM. cfGFP(hC) **12** was allowed to react with FM **14** to yield cfGFP(hC)-FM **16**.

6-FAM maleimide was purchased from Lumiprobe© (cat. # 44180). The 6-FAM maleimide **14** (1.8 eq., 0.5 mg in 500 µl DMSO, 1 nmol μl^{-1}, 0.5 µl) was added to cfGFP(hC) **12** (0.28 nmol/10 µl in PBS pH 7.4) and incubated at 37 °C for 30 min, w/up. The product **16** was analysed by ESI QToF MS. Calculated MW: 27137 Da; Detected MW: 27140 Da.

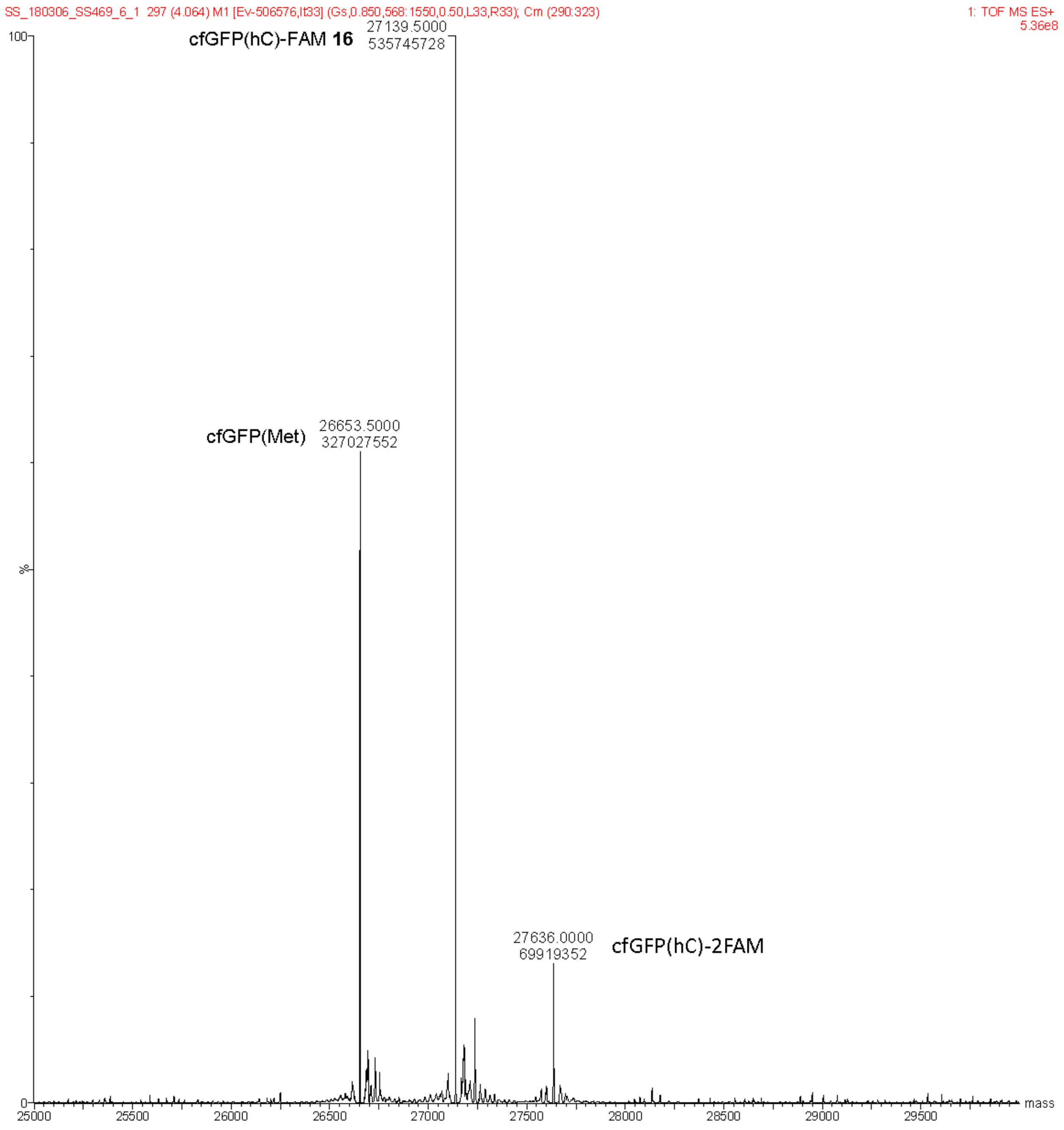

Figure S21. ESI QToF spectrum of FAM maleimide **14** conjugation with cfGFP(hC) **12** after 30 min.

*2.8. Conjugation of FAM maleimide **14** to fcGFPhs1-RM(2hC) **13***

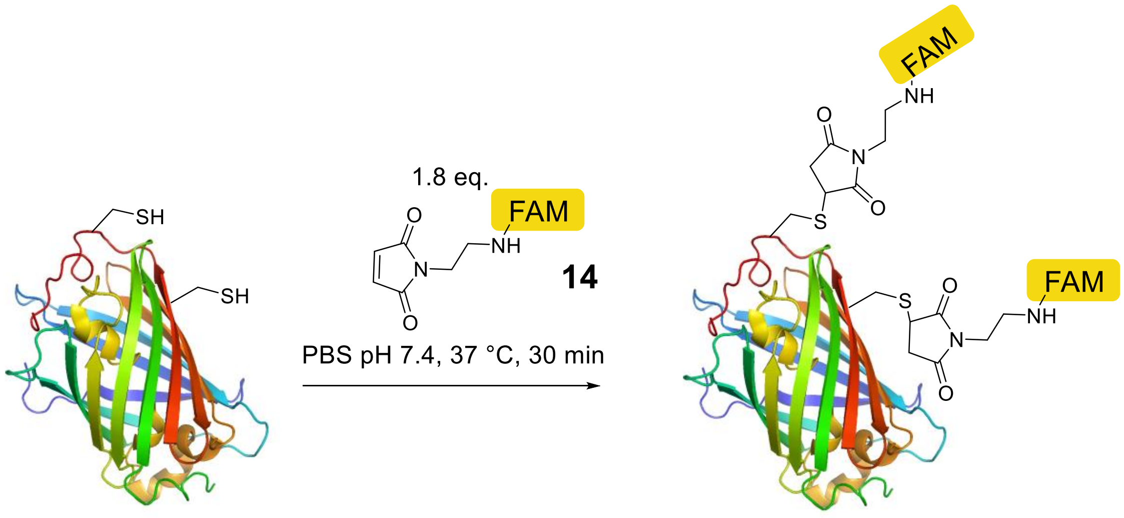

Figure S22. Conjugation reaction of fcGFPhs1-RM(2hC) with FM. cfGFP(2hC) **13** was allowed to react with FM **14** to yield cfGFP(2hC)-2FM **17**.

6-FAM maleimide was purchased from Lumiprobe (cat. # 44180). The 6-FAM maleimide **14** (1.8 eq., 0.5 mg in 500 µl DMSO, 1 nmol μl^{-1}, 0.5 µl) was added to cfGFP(2hC) **13** (0.28 nmol/10 µl in PBS pH 7.4) and incubated at 37 °C for 30 min, w/up. The product **17** was analysed by ESI QToF MS. Calculated MW: 27623 Da; Detected MW: 27627 Da.

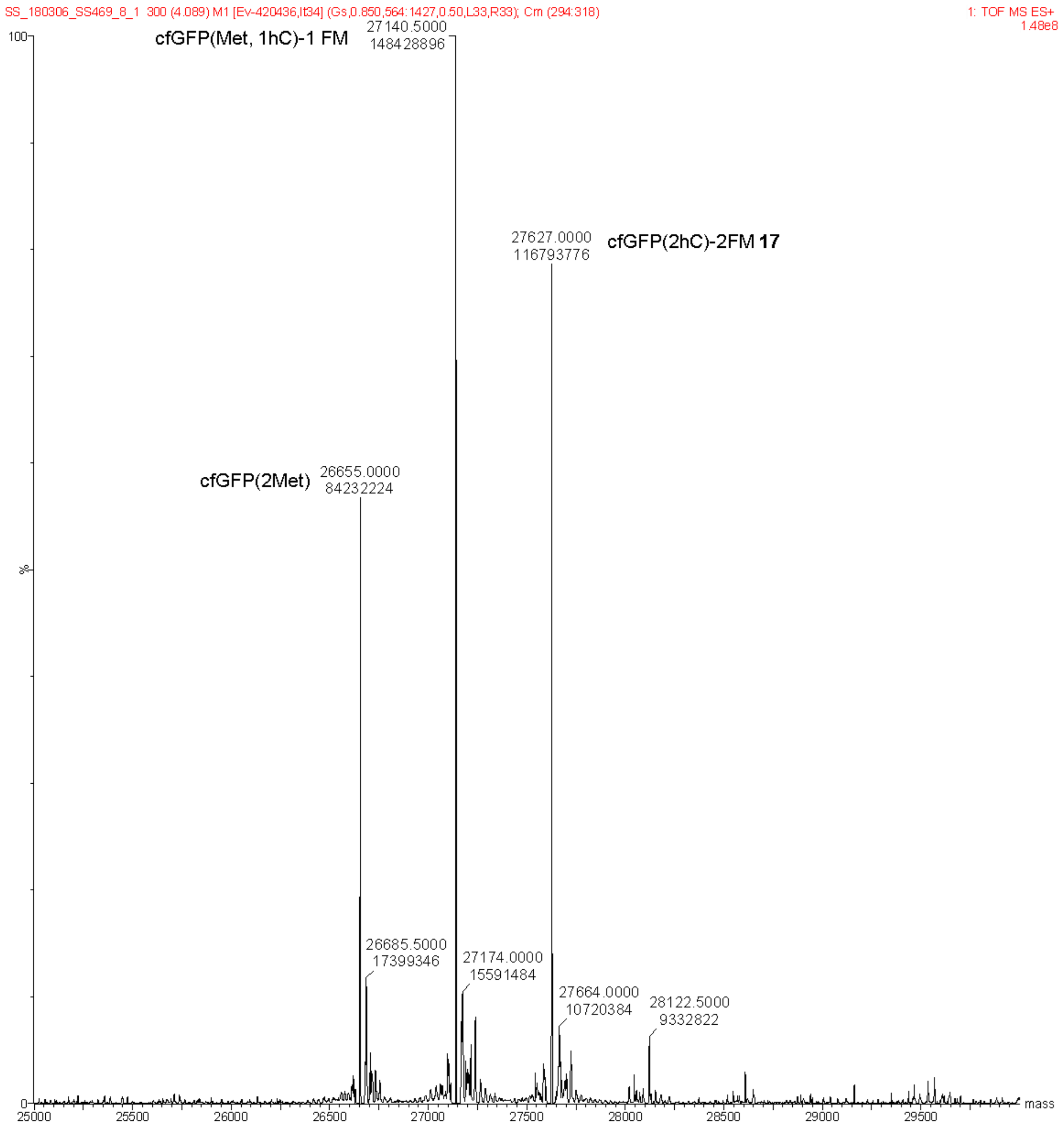

Figure S23. ESI QToF spectrum of FAM maleimide **14** conjugation with cfGFP(2 hC) **13** after 30 min.

*2.9. Conjugation of Phenyl phosphonamidate **15** to fcGFPhs1-RM(hC) **12***

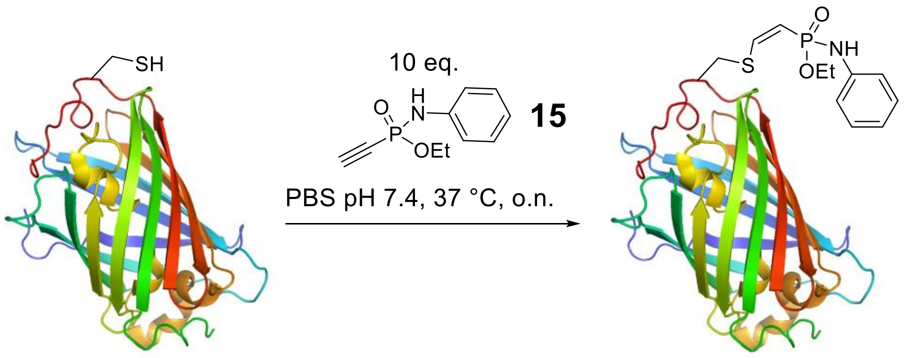

Figure S24. Conjugation reaction of cfGFPhs1-RM(hC) with PP. cfGFP(hC) **12** was allowed to react with PP **15** to yield cfGFPhs1-RM(hC)-PP **18**.

Phenyl phosphonamidate (PP) **15** (10 eq., 6 nmol µl^{-1}, 0.5 µl) was added to cfGFP(hC) **12** (0.28 nmol/10 µl in PBS pH 7.4) and incubated at 37 °C overnight, w/up. The product cfGFPhs1-RM(hC)-PP **18** was analysed by ESI QToF MS. Calculated MW: 2684 Da; Detected MW: 26848 Da.

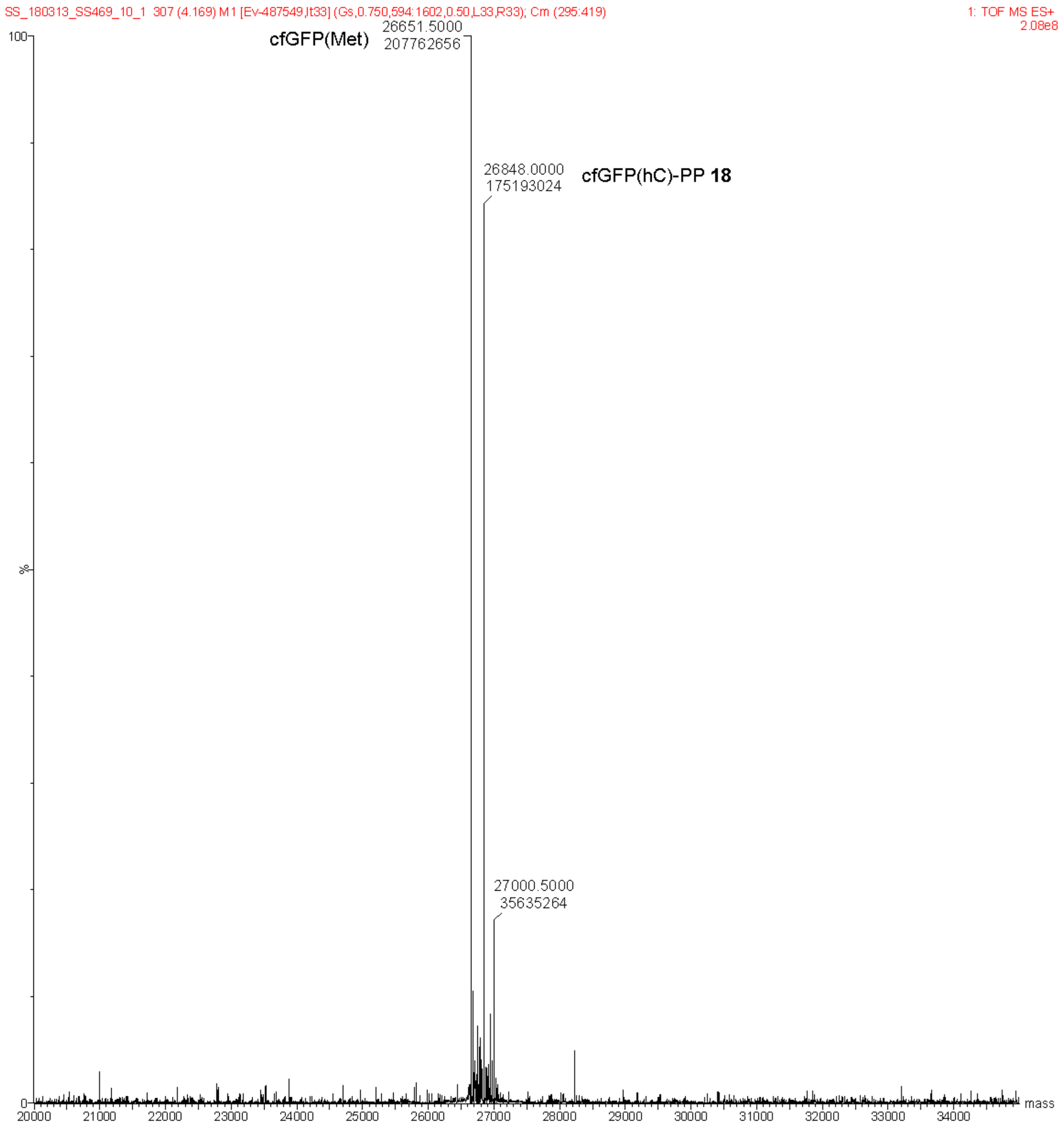

Figure S25. ESI QToF spectrum of Phenyl phosphonamidate (PP) **15** conjugation with cfGFP(hC) **12** after overnight.

2.10. Conjugation of Phenyl phosphonamidate to fcGFPhs1-RM(2hC) ***13***

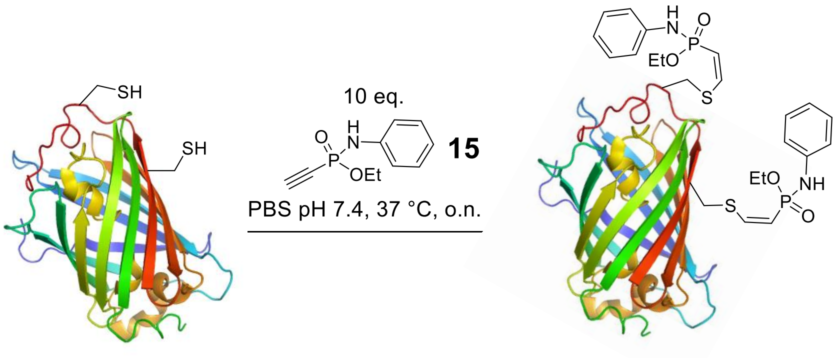

13 19

Figure S26. Conjugation reaction of cfGFPhs1-RM(2hC) with PP. cfGFPhs1-RM(2hC) **13** was allowed to react with PP **15** to yield cfGFPhs1-RM(2hC)-2PP **19**

Phenyl phosphonamidate (PP) **15** (10 eq., 6 nmol μl^{-1}, 0.5 μl) was added to cfGFPhs1-RM(2hC) **13** (0.28 nmol/10 μl in PBS pH 7.4) and incubated at 37 °C overnight, w/up. The product cfGFPhs1-RM(2hC)-2PP **19** was analysed by ESI QToF MS. Calculated MW: 27044 Da; Detected MW: 27045 Da.

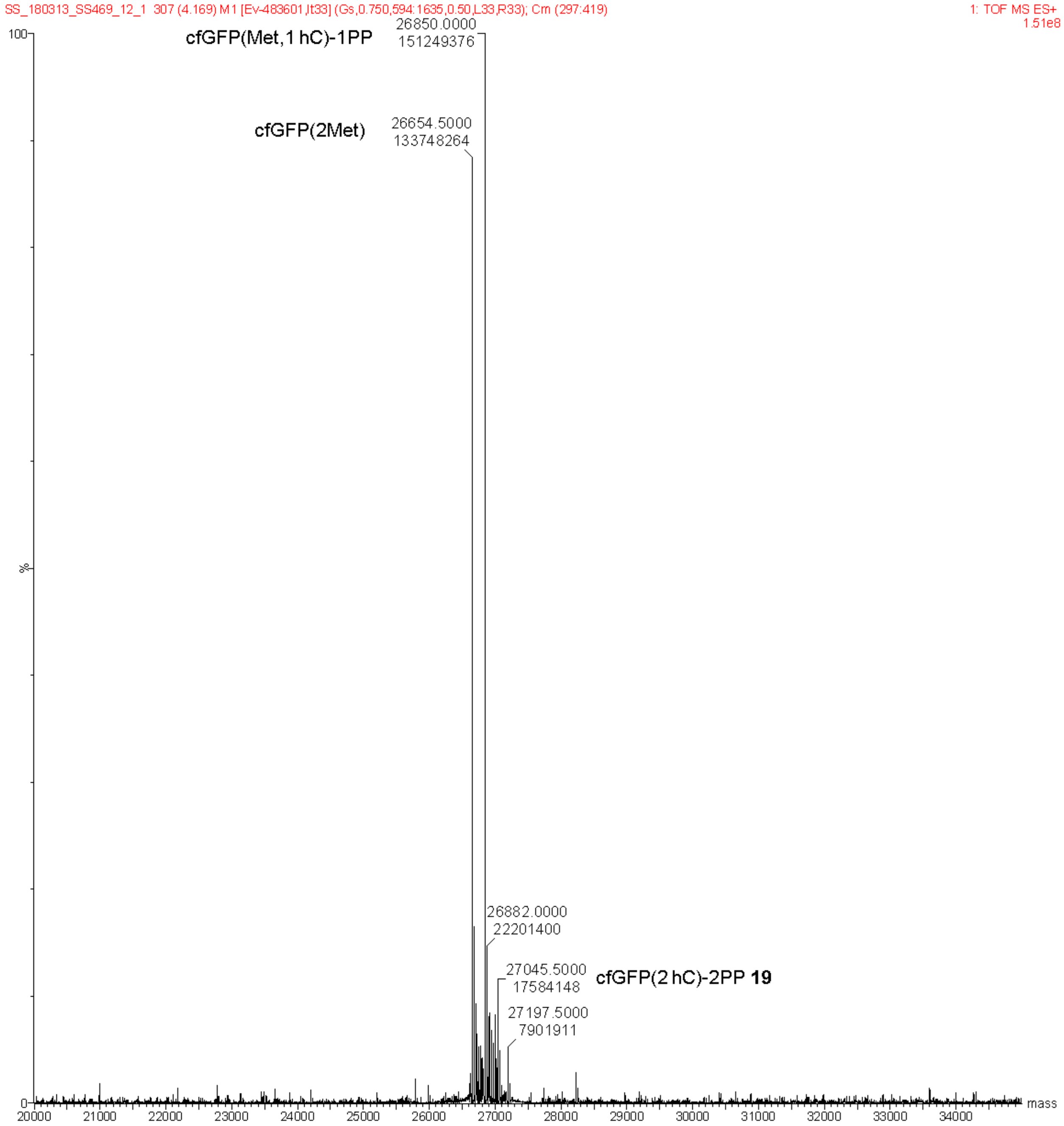

Figure S27. ESI QToF spectrum of Phenyl phosphonamidate (PP) **15** conjugation with cfGFPhs1-RM(2hC) **13** after overnight incubation.

Literature

1. Köhling, S.; Exner, M.P.; Nojoumi, S.; Schiller, J.; Budisa, N.; Rademann, J. One-Pot Synthesis of Unprotected Anomeric Glycosyl Thiols in Water for Glycan Ligation Reactions with Highly Functionalized Sugars. *Angew. Chem. Int. Ed. Engl.* **2016**, *55*, 15510–15514.

2. Kasper, M.-A.; Glanz, M.; Stengl, A.; Penkert, M.; Klenk, S.; Sauer, T.; Schumacher, D.; Helma, J.; Krause, E.; Cardoso, M.C.; et al. Cysteine-selective phosphonamidate electrophiles for modular protein bioconjugations. *Angew. Chem. Int. Ed. Engl.* **2019**, *In press,* (doi.org/10.1002/anie.201814715).
